# SGO enhanced random forest and extreme gradient boosting framework for heart disease prediction

**DOI:** 10.1038/s41598-025-02525-7

**Published:** 2025-05-25

**Authors:** Anima Naik, Ghanshyam G. Tejani, Seyed Jalaleddin Mousavirad

**Affiliations:** 1 Department of CSE, Raghu Engineering College, Visakhapatnam, Andhra Pradesh 530003 India; 2 Department of Research Analytics, Saveetha Dental College and Hospitals, Saveetha Institute of Medical and Technical Sciences, Saveetha University, Chennai, 600077 India; 3 Department of Industrial Engineering and Management, Yuan Ze University, Taoyuan, 320315 Taiwan; 4 Department of Computer and Electrical Engineering, Mid Sweden University, Sundsvall, 851 70 Sweden

**Keywords:** SGO, RF, XGBClassifier, Heart disease, Cleveland dataset, Statlog dataset, Applied mathematics, Computational science

## Abstract

Cardiovascular disease (CVD) remains a leading global health concern, accounting for approximately 31.5% of deaths worldwide. According to the World Health Organization (WHO), over 20.5 million people succumb to CVD each year—a figure projected to rise to 24.2 million by 2030. Early diagnosis is critical and can be facilitated by monitoring key risk factors such as cholesterol levels, blood pressure, diabetes, and obesity. This study proposes a heart disease prediction (HDP) model employing Random Forest (RF) and eXtreme Gradient Boosting (XGB) classifiers. Both models are further optimized through hyperparameter tuning using the Social Group Optimization (SGO) algorithm. The model was developed and validated using the Cleveland and Statlog datasets from the UCI repository. Pre-optimization results for RF yielded an accuracy (Acc.) of 84% and a ROC-AUC score of 92.03% on the Cleveland dataset, and 88.09% Acc. with a ROC-AUC of 97.50% on Statlog. The XGB classifier achieved 81.97% Acc. and a ROC-AUC of 90.73% on Cleveland, and 92.86% Acc. with a ROC-AUC of 96.14% on Statlog. After SGO-based optimization, RF improved to 95.08% Acc. and 95.26% ROC-AUC on Cleveland, and 95.24% Acc. with 98.18% ROC-AUC on Statlog. Similarly, the optimized XGB classifier reached 93.44% Acc. and 95.24% ROC-AUC on Cleveland, and 97.62% Acc. with 97.50% ROC-AUC on Statlog. These results highlight the effectiveness of SGO in enhancing ML performance for medical prediction problems. However, the study has certain limitations. The evaluation was conducted solely on two benchmark datasets, which may not fully reflect the diversity and complexity of real-world clinical populations. Furthermore, external validation using independent or real-time clinical data was not performed, which may limit the generalizability of the results. The computational cost associated with SGO optimization was also not assessed. Future research should focus on validating the model across broader datasets, assessing real-world applicability, and analyzing computational efficiency to ensure scalability and clinical adoption.

## Introduction

Recently, heart disease continues to be a leading cause of death worldwide, with significant societal and economic impacts. Following the World Health Organization (WHO) report of 2005, globally 17.5 million of 58 million deaths occurred due to heart disease (HD)^1^. Regardless of developed countries diminishing the mortality rate due to HD, the predominance remains high in developing countries, especially in India and other southern parts of Asia. Early and accurate detection of heart conditions is crucial for timely intervention and effective treatment. Recent advancements in cardiac healthcare have explored various technological and biomedical approaches to improve diagnosis and treatment outcomes. Innovations range from wearable and implantable devices^2^, heart sound representation learning^3^, to molecular mechanisms involved in cardiovascular disease progression and therapy^4–7^. Furthermore, machine learning and signal processing techniques have been applied to electrocardiogram (ECG) analysis^4^, cardiotoxicity detection^5,6^, cardiac fibrosis regulation^7^, and drug-related adverse cardiovascular outcomes^8^. Studies have also highlighted the utility of novel 4D printed devices^9^, and deep learning (DL) models for vascular interventions^10^. These findings reinforce the importance of computational models and intelligent systems in modern cardiology^7,8,11–13^, paving the way for AI-augmented predictive tools in healthcare decision-making. Traditional diagnostic methods, while valuable, often rely heavily on the expertise of clinicians and can be subject to variability and limitations in sensitivity. In this context, machine learning (ML) algorithms have emerged as transformative tools, offering the ability to analyze complex and high-dimensional datasets with remarkable efficiency and Acc. The evolution of artificial intelligence (AI) and ML in diverse areas^14,15^ affords genuine benefits in predicting HDs. Among these, ensemble methods such as Random Forest (RF) and XGB Classifier have gained widespread recognition for their robustness and predictive performance^16^.

Despite the potential of ML algorithms in medical diagnostics, their performance is not solely determined by the choice of the model. Hyperparameter tuning is essential for shaping a model’s performance, as it directly influences Acc, interpretability, and generalizability. A predictive model’s level of complexity and fit is determined by important hyperparameters, including the number of trees in a forest, the maximum depth of decision trees, and the learning rate of boosting models^17^. However, the large and frequently nonlinear search space makes it difficult to find the ideal combination of these hyperparameters. The predictive ability of even the most sophisticated models can be limited by poorly selected configurations that cause overfitting or underfitting^18^.

Conventional hyperparameter tuning techniques like grid search and random search are simple to use but frequently have efficiency issues, particularly when working with big or complicated search spaces. These techniques are less useful for datasets with many features and complex hyperparameter interactions because they are not well suited to prioritizing the most promising regions. In order to get around these restrictions, scientists have resorted to sophisticated optimization strategies like metaheuristic algorithms, which take cues from natural and evolutionary processes to more successfully traverse challenging search environments^19^. Inspired by biological and natural processes, metaheuristic algorithms have shown themselves to be effective tools for hyperparameter optimization. A method that has garnered a lot of attention is SGO^20,21^, which is based on human social behavior and interactions. Through the use of both individual experiences and group knowledge, SGO mimics how members of a social group learn and adjust. SGO is especially well-suited for handling challenging nonlinear optimization problems in ML because of its dual learning mechanism, which efficiently strikes a balance between exploration and exploitation.

Because SGO is independent of derivative information - unlike conventional optimization algorithms - it is particularly well-suited for black-box optimization problems involving non-differentiable or discontinuous objective functions. Table 1 provides a summary of recent studies that demonstrate the versatility and effectiveness of the SGO algorithm across a wide range of application domains, including engineering design, medical diagnosis, image segmentation, and wireless networks. These studies collectively highlight SGO’s robust exploration-exploitation balance, adaptability, and computational stability, which justify its selection in our study for hyperparameter tuning of classifiers.

**Table 1:** Overview of applications of the SGO algorithm.

Study	SGO variant	Application domain	Key findings
Naik et al.^22^	SGO	Multimodal function optimization & clustering	Demonstrated strong global search capability
Naik et al.^23^	Binary SGO	0–1 Knapsack problem	Outperformed other binary optimizers
Monisha et al.^24^	SGO + Shannon Function	RGB image thresholding	Achieved optimal segmentation results
Reddy et al.^25^	ME-SGO	Engineering and EV optimization	Overcame standard SGO limitations
Manic et al.^26^	SGO + TSALLIS Entropy	Brain MRI segmentation	Improved detection efficiency
Parwekar et al.^27^	SGO	Energy optimization in WSNs	Reduced transmission distance and energy use
Das et al.^28^	MSGO	Damage detection in civil structures	Improved damage identification accuracy
Satapathy et al.^29^	NS-SGO	Multi-objective optimization	Competitive results with other methods
Naik^30^	Chaotic SGO	Structural engineering problems	Solved both unimodal and multimodal tasks
Naik^31^	Marine Predators + SGO	Hybrid metaheuristics	Enhanced marine predator optimization
Naik^32^	Multi-objective SGO	Machining process	Improved efficiency through Pareto optimization
Dey et al.^33^	SGO-assisted Kapur Entropy	COVID-19 CT segmentation	Boosted segmentation accuracy
Singh et al.^34^	SGO + SVC	COVID-19 X-ray classification	Enhanced diagnostic performance
Akhtar et al.^35^	DESGO	Inventory optimization	Handled deteriorating items effectively
Kalananda et al.^36^	SGWOA	Engineering optimization	Outperformed standard WOA
Kraiem et al.^37^	Modified SGO	PV cell parameter tuning	Accurate model identification
Secui et al.^38^	Modified SGO	Wind-integrated emission dispatch	Achieved optimal dispatch scheduling
Meesala et al.^39^	V/S-MSGO	Feature selection	Found minimal feature sets
Meesala et al.^40^	Auto-SGO	Clustering & feature selection	Optimal clusters with adaptive thresholds
Naik^41^	Enhanced SGO	Global optimization	Applied to structural engineering tasks
Tran et al.^42^	MO-SGO + MCDM	Construction cost-time tradeoff	Effective multi-criteria optimization
Fang et al.^43^	SGO + SVM	Transformer fault diagnosis	Improved fault detection
Huynh et al.^44^	MO-SGO	Construction project planning	Optimized time, cost, quality, and emissions
Vadivel et al.^45^	SGO-assisted MPPT	PV power tracking	Improved tracking under partial shading
Wang et al.^46^	Dual-Population SGO	Metaheuristic optimization	Enhanced global convergence
Tran et al.^47^	SGO + Smeared Stiffener	Panel frequency optimization	Achieved maximum frequency in structures
Garg & Kishore^48^	SGO	Image watermarking	Developed a secure adaptive method
Rahaman et al.^49^	SGO	UAV charging schedule	Efficient scheduling for sensor networks

Table 1 presents a comprehensive overview of various SGO variants and their applications. For instance, Naik et al.^22,23^ applied SGO and Binary SGO to clustering and combinatorial problems with superior performance, while Monisha et al.^24^ and Manic et al.^26^ used SGO-based techniques for image and brain MRI segmentation, achieving optimal results. In wireless sensor networks and energy optimization, Parwekar et al.^27^ demonstrated reduced energy consumption using SGO. Moreover, hybrid and enhanced SGO versions such as ME-SGO, Chaotic SGO, and Auto-SGO have achieved state-of-the-art performance in structural engineering, inventory management, and feature selection^25,30,40^.

Based on these results, SGO has consistently delivered competitive or superior outcomes, making it a strong candidate for optimizing ML models in HDP. Although several metaheuristic algorithms such as Genetic Algorithm (GA), Particle Swarm Optimization (PSO), and Differential Evolution (DE) are commonly used for hyperparameter tuning, SGO was selected in our study due to its superior balance between exploration and exploitation, minimal sensitivity to parameter settings, and consistent robustness across diverse optimization problems.

Unlike GA and PSO, which may suffer from premature convergence or require significant tuning of control parameters, SGO mimics human social behavior to dynamically guide the search process. This reduces the likelihood of stagnating in local optima and allows the algorithm to maintain a good balance between convergence speed and global search. Additionally, SGO has demonstrated strong performance in both unimodal and multimodal optimization scenarios, as summarized in Table 1. These advantages underscore the suitability of SGO for hyperparameter tuning in medical classification tasks such as HDP. This work extends the use of SGO to RF and XGB classifier hyperparameter tuning, particularly for the prediction of heart disease. Cleveland Heart Disease^50^ and Statlog Heart Disease^51^ are two well-known benchmark datasets that we use to assess the efficacy of the suggested methodology. Because of their wide range of features and practicality, these datasets are frequently utilized in medical predictive analytics. Our goal in using SGO is to improve predictive Acc. by fine-tuning the RF and XGB hyperparameters. while maintaining the effectiveness of computation.

Because of their unique benefits, RF and XGB were chosen as classifiers. Even with little parameter adjustment, RF, an ensemble of decision trees, performs well and is very resistant to overfitting. By comparison, XGB is a strong gradient-boosting framework that is renowned for its remarkable Acc. as well as flexibility, particularly when working with unbalanced datasets. Combining these classifiers gives us a strong basis for predicting HD. Through precise hyperparameter tuning with SGO, their performance can be further improved, leading to better predictive Acc. as well as effectiveness^52^.

This research paper suggests the following contributions.

- Advancement in Metaheuristic Optimization: The research demonstrates the practical application of the SGO algorithm in hyperparameter tuning, contributing to the growing body of work on metaheuristic optimization techniques in ML.
- Creative Algorithm Integration: The study presents a novel strategy by fusing the predictive capabilities of RF and XGB Classifier with the effectiveness of SGO. This combined strategy successfully addresses current healthcare issues, particularly with regard to heart disease prediction.
- Generalizability Through Comparison: The robustness and adaptability of the suggested approach are confirmed by a comprehensive comparison analysis utilizing two distinct datasets. The findings provide insightful information for applying ML models in a range of clinical settings.
- Scalable Framework for Healthcare Applications: By creating an efficient and scalable framework for hyperparameter tuning, this study paves the way for applying SGO and similar algorithms across different areas of healthcare.
- Broader Implications for Predictive Analytics: This research fosters innovation in predictive analytics and decision support systems, offering a path for improved performance in ML applications beyond heart disease prediction.

The structure of this paper is as follows: Sect. 2 reviews related work, Sect. 3 details the SGO algorithm, Sect. 4 introduces the methodology, Sect. 5 describes the proposed SGO-optimized RF algorithm, Sect. 6 presents the experimental design, results analysis, and discussions, Sect. 7 presents generalizability of the proposed SGO-tuned ML Model and Sect. 8 presents conclusions and future research directions.

## Related work

Over the past decade, numerous studies have been conducted on heart disease prediction using ML and metaheuristic techniques. Researchers have explored a wide range of classifiers - from traditional algorithms like logistic regression (LR) and decision trees (DTs) to more advanced models such as DL, ensemble methods, and optimization-based hybrids. Various datasets, particularly the Cleveland and Statlog UCI datasets, have served as benchmarks for these evaluations. Table 2 summarizes key contributions from existing literature, highlighting the methods used, datasets employed, Acc. and performance metrics achieved, and notable remarks on their effectiveness.

**Table 2:** Summary of related work on HDP using ML and optimization techniques.

Author(s)	Method/algorithm	Dataset(s)	Key results (Acc., Prec., Rec., F1, etc.)	Remarks
Premsmith et al.^53^	Logistic Regression, Neural Networks	UCI Heart Dataset	Acc: 91.65%, Prec: 95.45%, Rec: 84%, F1: 89.36%	Logistic regression outperformed neural networks
Latha et al.^54^	Ensemble (Bagging, Boosting)	UCI Heart Dataset	~ 7% Acc. improvement for weak classifiers	Ensemble methods enhanced weak classifiers
Chaurasia et al.^55^	Naive Bayes, J48, Bagging	UCI Heart Dataset	Best Acc: 85.03% (Bagging)	Bagging yielded the best results
Mienye et al.^56^	PSO + SSAE	UCI Heart Dataset	Acc: 96.1%, Prec: 93.0%, Sensitivity: 98.8%	Strong performance with swarm-based DL
Batainh et al.^57^	PSO + MLP	UCI Heart Dataset	Acc: 84.6%	Hybrid method showed promising performance
Bhatt et al.^16^	Multiple ML Algorithms (including MLP)	Kaggle Heart Dataset	Acc: 87.28% (MLP highest)	Comprehensive algorithm comparison
Khan et al.^58^	RF, LR, DT, Naive Bayes	UCI Heart Dataset	Acc: 85.01% (RF highest)	RF outperformed other classifiers
Ahmad et al.^59^	Stacked Cross-Validation Classifier	Cleveland + other datasets	Enhanced Acc. via feature-risk analysis	Established correlation between risk factors and prediction
Mohan et al.^60^	Hybrid RF + Linear Model (HRFLM)	Cleveland Dataset	Improved Acc.	Combined linear models with RF for better prediction
Noroozi et al.^61^	Naive Bayes, J48, Bagging	Cleveland Dataset	NB: 82.31%, J48: 84.35%, Bagging: 85.03%	Bagging performed best
Reddy et al.^62^	RF for Feature Selection + Classification	Combined datasets (Cleveland, Statlog, Hungary, etc.)	Acc: 90–95%	Merged datasets for broader coverage
Khan et al.^63^	DT (C4.5), NB, SVM, RIPPER, KNN, GA, ANN	Cleveland Dataset	DT, NB, and SVM outperformed others	Comprehensive comparison of classifiers
Ahmed et al.^64^	SVM, RF, LR, DT + Feature Selection	Cleveland + Social Media Data	Acc: 94–99% (RF highest)	Used big data + social media + feature selection
Latha & Jeeva^65^	Boosting & Bagging + PSO	Cleveland Dataset	Acc. improvement up to 7%	Enhanced weak classifiers using ensemble + PSO
Ahmad & Polat^66^	SVM + JOT	Small UCI Dataset	Acc: 98.47%	Strong results but limited by dataset size
Chandrashekar & Narayanreddy^67^	ETHO	Not specified	Acc: 98.61%	High Acc., but limited generalizability
Sun & Pan^68^	LR, KNN, DT, SVM, RF + Health Indicators	Self-measurable Indicators	Up to 19.67% improvement	Good with full indicators, less Acc. with limited input
Jawalkar et al.^69^	DT-based RF + SGB Optimization	Not specified	Acc: 96.00%	High Acc. but computationally expensive
Srinivasan et al.^70^	LVQ	Not specified	Acc: 98.70%	Accurate but complex and resource-intensive
Vardhan et al.^71^	EM with DT, NB, LR, RF, SVM	Specific UCI Datasets	Significant Acc. improvement	Effective but dataset-dependent

The review of existing approaches reveals that while several traditional and ensemble-based methods have achieved commendable results, challenges such as overfitting, limited generalizability, and computational complexity persist. Notably, optimization techniques like PSO and ensemble learning have significantly improved classification performance. However, there remains a gap in employing more recent metaheuristic algorithms for hyperparameter tuning in predictive models. To address these limitations, this study proposes the use of the SGO algorithm for tuning RF and XGB classifiers. The objective is to enhance prediction Acc. and model robustness while maintaining computational efficiency across standard HD datasets. Such studies highlight the significance of hyperparameter tuning and algorithm selection for the prediction of HD. State of the art techniques keep proving their worth in enhancing diagnostic Acc. and effectiveness assisting medical practitioners in producing better results.

## SGO algorithm

Suresh Satapathy et al. developed the SGO algorithm^20^. is an optimization method that draws inspiration from human social interactions and learning patterns. The Improving Phase and the Acquiring Phase are the two main stages it uses to address optimization issues. People hone their solutions during these stages by talking with other group members and picking up tips from the top performers. This dynamic process makes SGO an efficient optimization technique by ensuring a balanced approach between exploration (looking for new solutions) and exploitation (improving on ones that already exist).

a Improving phase.
  The Improving Phase is focused on self-improvement where each person learns from the groups best performer to improve their own solution. Convergence toward ideal solutions is largely driven by this stage.
  1 $$\:{X}_{i}^{new}=C.{X}_{i}^{old}+{r}_{1}\cdot\:({X}_{best}-{X}_{i}^{old})$$
  Where:
  $\:{X}_{i}^{new}$: Updated position of the i-th individual.
  $\:{X}_{i}^{old}$: Previous position of the i-th individual.
  $\:{X}_{best}$: Best solution found in the population.
  $\:C$: Self-introspection parameter (a constant between 0 and 1).
  $\:{r}_{1}$: Random number in [0,1], introducing controlled randomness.
  During this stage the global best solution is taken into account when adjusting each persons position. Self-reflection parameter C regulates the rate of learning and the random factor $\:{r}_{1}$ guarantees search space exploration. The population is led toward the ideal solution during this phase.
b. Acquiring phase.
  The Acquiring Phase models the interaction between individuals. Each individual compares its fitness with that of a randomly chosen peer and updates its position based on the peer’s position and the global best solution. This phase ensures both exploitation and diversity.
  2 $$\:{X}_{i}^{new}=\:\left\{\begin{array}{c}{X}_{i}^{old}+{r}_{2}.\left({X}_{i}-{X}_{k}\right)+{r}_{3}.\left({X}_{best}-{X}_{i}\right)\:\:\:if\:f\left({X}_{i}\right)<f\left({X}_{k}\right)\\\:{X}_{i}^{old}+{r}_{2}.\left({X}_{k}-{X}_{i}\right)+{r}_{3}.\left({X}_{best}-{X}_{i}\right)\:\:\:\:\:otherwise\:\:\:\:\:\:\:\:\:\:\:\end{array}\right.$$
  Where:
  $\:{X}_{i}$ : Current position of the i-th individual.
  $\:{X}_{k}$: Position of a randomly selected k-th individual $\:(k\ne\:i)$
  $\:{X}_{best}$: Best solution found in the population.
  $\:f\left({X}_{i}\:\right)$ and $\:f\left({X}_{k}\:\right)$: Fitness values of the i-th and k-th individuals, respectively.
  $\:{r}_{2}$, $\:{r}_{3}$: Random numbers in [0,1] that introduce stochastic behavior.

Each person engages with a peer ($\:{X}_{k}\:$) who is chosen at random during this phase. The update is driven by the difference between their positions and the global best solution if the individual’s fitness $\:f\left({X}_{i}\:\right)$ is superior to that of its peer $\:f\left({X}_{k}\:\right)$. However, if the peer has a better fitness value, the individual moves closer to both the peer and the global best, improving its chances of finding an optimal solution. This phase reinforces the influence of high-quality solutions while maintaining diversity.

*Algorithm steps*.

1. Initialization:
  Define the population size (N), maximum iterations MaxIter, and the search space bounds (LB, UB).
  Randomly initialize N individuals within the bounds and evaluate their fitness using the objective function.
  Identify the global best solution $\:{X}_{best}$.
2. Repeat Until Stopping Criteria are Met:
  Improving Phase: Update each individual’s position using self-learning based on the global best solution.
  Acquiring Phase: Update each individual’s position through interaction with another randomly selected individual and the global best solution.
  Evaluate the new fitness values and update the global best solution $\:{X}_{best}$.
3. Return the best solution.
  Output the global best solution and its corresponding fitness value.

**Algorithm 1:**
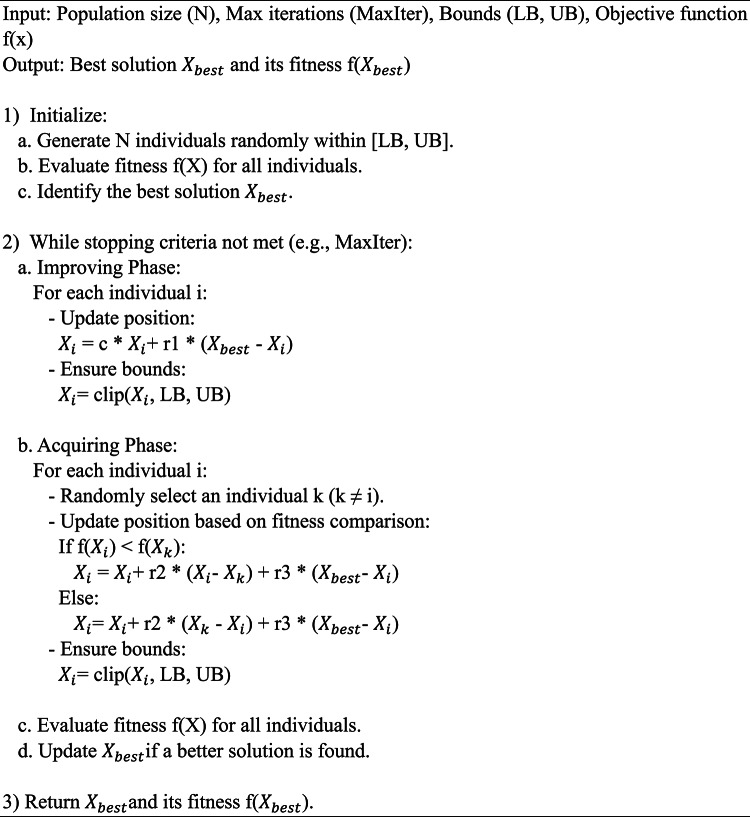
Pseudocode of SGO.

Key features of SGO.

- Exploration and Exploitation: The Improving Phase emphasizes convergence, while the Acquiring Phase maintains diversity through peer interaction.
- Simplicity: The algorithm’s design is straightforward, requiring minimal parameter tuning.
- Global Search Capability: By learning from the global best and peer interactions, SGO effectively avoids local optima.

## Materials and methods

### Global overview of the proposed methodology

**Fig. 1:**
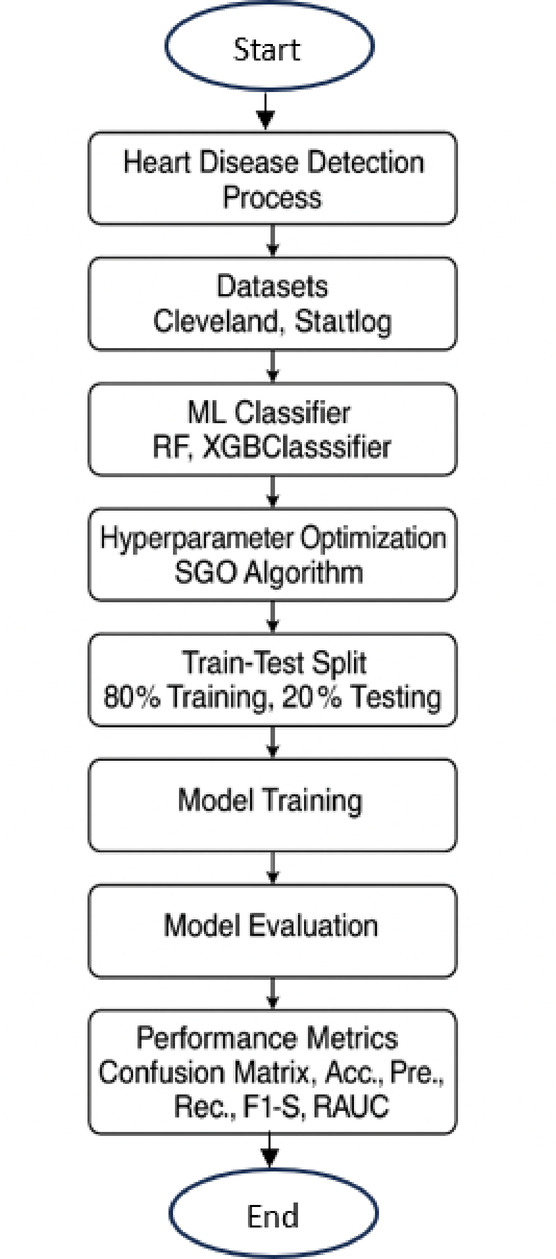
Flow diagram for heart disease detection process.

The primary goal of this experiment is to identify an effective and accurate algorithm for detecting heart diseases, as outlined in Fig. 1. To achieve this, we employed a ML classifier and optimized its hyperparameters using the SGO metaheuristic algorithm. The objective of this optimization is to determine the best parameter set to enhance the model’s Acc. in predicting heart disease. We used RF and XGB Classifier as classifiers, and the datasets utilized were the Cleveland and Statlog datasets, which are processed datasets derived from Kaggle. RF and XGB were chosen due to their proven effectiveness in handling high-dimensional, imbalanced, and noisy datasets - characteristics commonly found in medical data such as heart disease records. RF is known for its robustness, ability to reduce overfitting through ensemble learning, and interpretability. XGB, on the other hand, offers superior performance through gradient boosting and efficient handling of complex feature interactions. Both classifiers have demonstrated strong predictive capabilities in previous healthcare-related studies, making them suitable and reliable choices for HDP.

Before training the models, we first tuned their hyperparameters using the SGO algorithm to find the optimal configuration. To evaluate the performance of the models, we split the dataset into 80% for training and 20% for testing using the train_test_split method. The models were trained on the training set (80%) and evaluated on the test set (20%) to assess their predictive Acc. After testing, we generated a confusion matrix and calculated various performance metrics, including Acc., Prec., Rec., F1-S, and RAUC score, to comprehensively evaluate the effectiveness of the models.

### Description of datasets used in this research

In this research work Cleveland and Statlog Heart Disease dataset have been utilized. The Cleveland^50^ and Statlog^51^ datasets were selected for this study due to their widespread acceptance and extensive use as benchmark datasets in HDP research. Both datasets originate from the UCI ML Repository and are considered reliable standards for evaluating classification models in the medical domain. The Cleveland dataset comprises 303 patient records with 14 clinical attributes. In contrast, the Statlog dataset is a preprocessed version of the Cleveland dataset, containing 270 instances with the same feature set but with missing values and outliers removed. The Statlog version offers a cleaner dataset, making it suitable for model evaluation without extensive data preprocessing. Using both datasets allows for a comprehensive assessment of the model’s robustness and generalizability across data with varying levels of noise and completeness. Their inclusion enhances the credibility of experimental results and supports meaningful comparison with existing methods in the literature.

#### Cleveland heart disease dataset

The Cleveland HD dataset^50^ is one of the most widely used datasets in ML and medical research for predicting heart disease. This dataset includes patient data such as demographic, clinical, and diagnostic attributes that can help predict the presence of heart disease. We collected this dataset from Kaggle.

The Cleveland dataset contains 303 instances and 14 attributes (including the target variable), including both continuous and categorical variables. The features represent various clinical measurements and results that could be useful in predicting heart disease:

- Age: The age of the patient (continuous).
- Sex: Gender of the patient (binary: 0 = female, 1 = male).
- Chest Pain Type (cp.): Type of chest pain experienced by the patient (categorical: 1 to 4, representing different types of pain).
- Resting Blood Pressure (trestbps): Blood pressure measured in mm Hg.
- Serum Cholesterol (chol): Serum cholesterol in mg/dl.
- Fasting Blood Sugar (fbs): Fasting blood sugar > 120 mg/dl (binary: 0 = no, 1 = yes).
- Resting Electrocardiographic Results (restecg): Electrocardiographic results (categorical: 0, 1, 2).
- Maximum Heart Rate Achieved (thalach): The maximum heart rate achieved during exercise.
- Exercise Induced Angina (exang): Whether the patient experiences exercise-induced angina (binary: 0 = no, 1 = yes).
- Oldpeak: Depression induced by exercise relative to rest (continuous).
- Slope of the Peak Exercise ST Segment (slope): Slope of the peak exercise ST segment (categorical: 1, 2, 3).
- Number of Major Vessels Colored by Fluoroscopy (ca.): Number of major vessels colored by fluoroscopy (categorical: 0 to 3).
- Thalassemia (thal): Thalassemia (categorical: 3 = normal, 6 = fixed defect, 7 = reversible defect).
- Target: The presence of heart disease (binary classification: 0 = no disease, 1 = presence of disease).

Cleveland dataset is often used for classification tasks, where the goal is to predict the target variable based on the other features. Table 3 provides the description of the dataset.

**Table 3:** Description of Cleveland dataset.

Index	Age	Sex	cp.	trestbps	chol	fbs	restecg	thalach	exang	oldpeak	Slope	ca.	thal	Target
count	303	303	303	303	303	303	303	303	303	303	303	303	303	303
mean	54.37	0.68	0.97	131.62	246.26	0.15	0.53	149.65	0.33	1.04	1.4	0.73	2.31	0.54
std	9.08	0.47	1.03	17.54	51.83	0.36	0.53	22.91	0.47	1.16	0.62	1.02	0.61	0.5
min	29	0	0	94	126	0	0	71	0	0	0	0	0	0
25%	47.5	0	0	120	211	0	0	133.5	0	0	1	0	2	0
50%	55	1	1	130	240	0	1	153	0	0.8	1	0	2	1
75%	61	1	2	140	274.5	0	1	166	1	1.6	2	1	3	1
max	77	1	3	200	564	1	2	202	1	6.2	2	4	3	1

#### Statlog heart disease dataset

The Statlog HD dataset^51^ is another widely used dataset for heart disease prediction. It contains 270 instances and 14 attributes (including the target variable). We collected this dataset from Kaggle. Like the Cleveland dataset, the features are clinical measurements related to heart disease diagnosis:

- Age: The age of the patient.
- Sex: Gender of the patient (binary: 0 = female, 1 = male).
- Chest Pain Type (cp.): Type of chest pain (categorical: 1 to 4).
- Resting Blood Pressure (trestbps): Blood pressure in mm Hg.
- Serum Cholesterol (chol): Serum cholesterol in mg/dl.
- Fasting Blood Sugar (fbs): Fasting blood sugar > 120 mg/dl (binary: 0 = no, 1 = yes).
- Resting Electrocardiographic Results (restecg): Electrocardiogram results (categorical: 0, 1, 2).
- Maximum Heart Rate Achieved (thalach): Maximum heart rate achieved during exercise.
- Exercise Induced Angina (exang): Whether exercise induces angina (binary: 0 = no, 1 = yes).
- Oldpeak: Depression induced by exercise.
- Slope of the Peak Exercise ST Segment (slope): Slope of the peak exercise ST segment (categorical: 1, 2, 3).
- Number of Major Vessels Colored by Fluoroscopy (ca.): Number of major vessels colored by fluoroscopy (categorical: 0 to 3).
- Thalassemia (thal): Type of thalassemia (categorical: 3 = normal, 6 = fixed defect, 7 = reversible defect).
- Target: The presence of heart disease (binary classification: 0 = no disease, 1 = presence of disease).

The Statlog dataset is typically used for binary classification tasks, where the goal is to predict whether a patient has heart disease based on various medical features. Table 4 provides the description of the dataset.

**Table 4:** Description of Statlog data.

Index	Age	Sex	cp.	trestbps	chol	fbs	restecg	thalach	exang	oldpeak	Slope	ca.	thal	Presence
count	270	270	270	270	270	270	270	270	270	270	270	270	270	270
mean	54.43	0.68	3.17	131.34	249.66	0.15	1.02	149.68	0.33	1.05	1.59	0.67	4.7	1.44
std	9.11	0.47	0.95	17.86	51.69	0.36	1	23.17	0.47	1.15	0.61	0.94	1.94	0.5
min	29	0	1	94	126	0	0	71	0	0	1	0	3	1
25%	48	0	3	120	213	0	0	133	0	0	1	0	3	1
50%	55	1	3	130	245	0	2	153.5	0	0.8	2	0	3	1
75%	61	1	4	140	280	0	2	166	1	1.6	2	1	7	2
max	77	1	4	200	564	1	2	202	1	6.2	3	3	7	2

### Data preprocessing

**Fig. 2:**
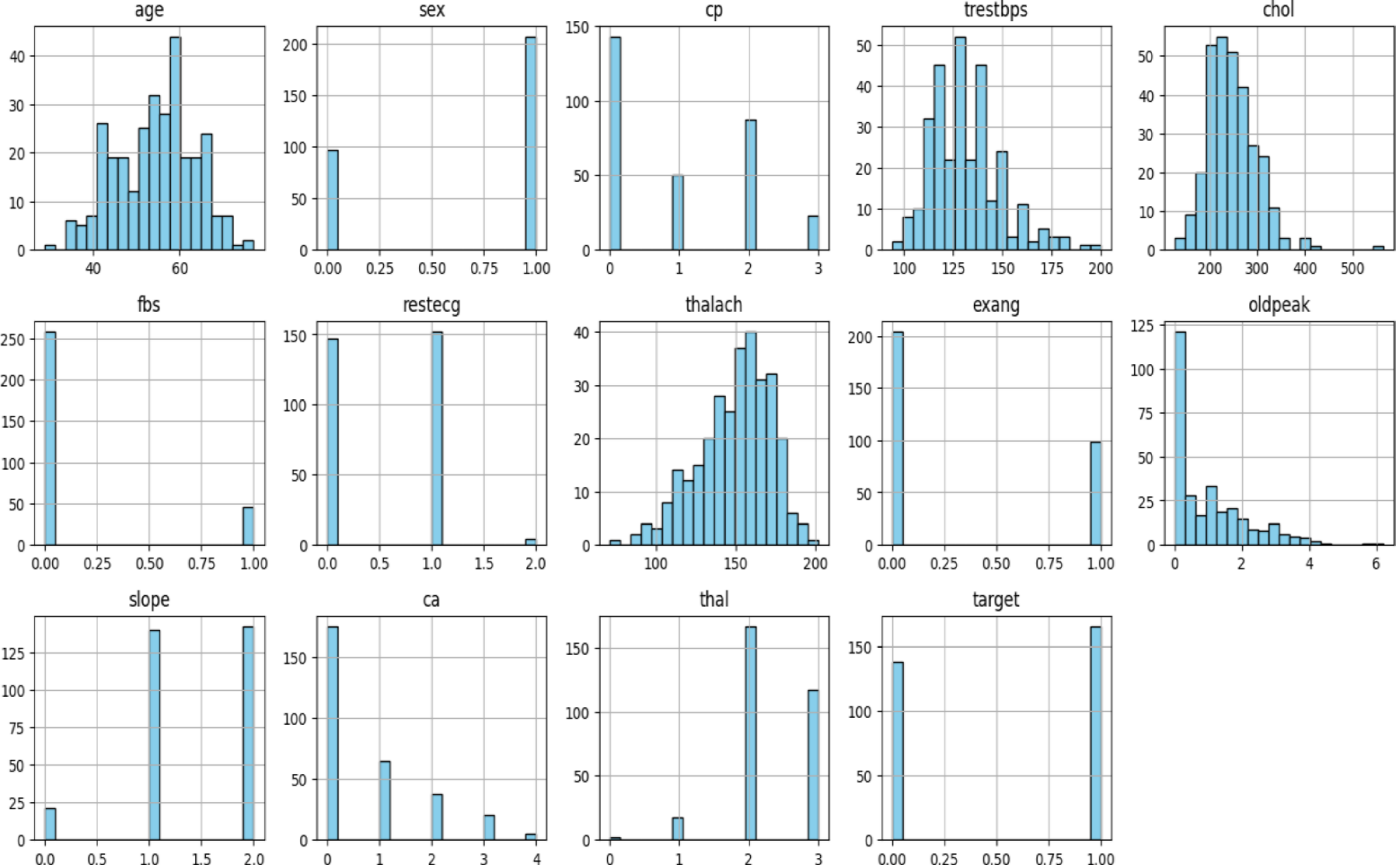
Histogram plot for each feature of Cleveland Heart Disease dataset.

We began the preprocessing phase by detecting and addressing missing values. The next step involved identifying and handling outliers. The goal was to identify data points that significantly deviate from the majority of the dataset. Various methods are available for detecting outliers, including the Z-score, Interquartile Range (IQR), and modified Z-score methods. The choice of method depends on the nature of the data, so selecting the appropriate technique is crucial (Fig. 2).

**Fig. 3:**
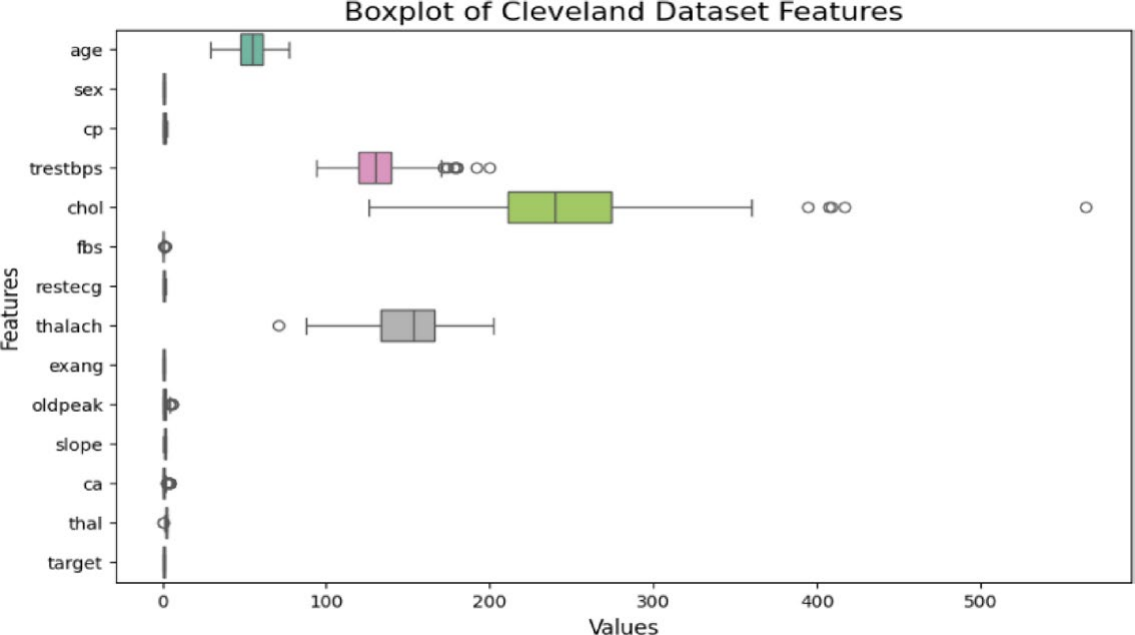
Box plot for each feature of Cleveland Heart Disease dataset.

**Fig. 4:**
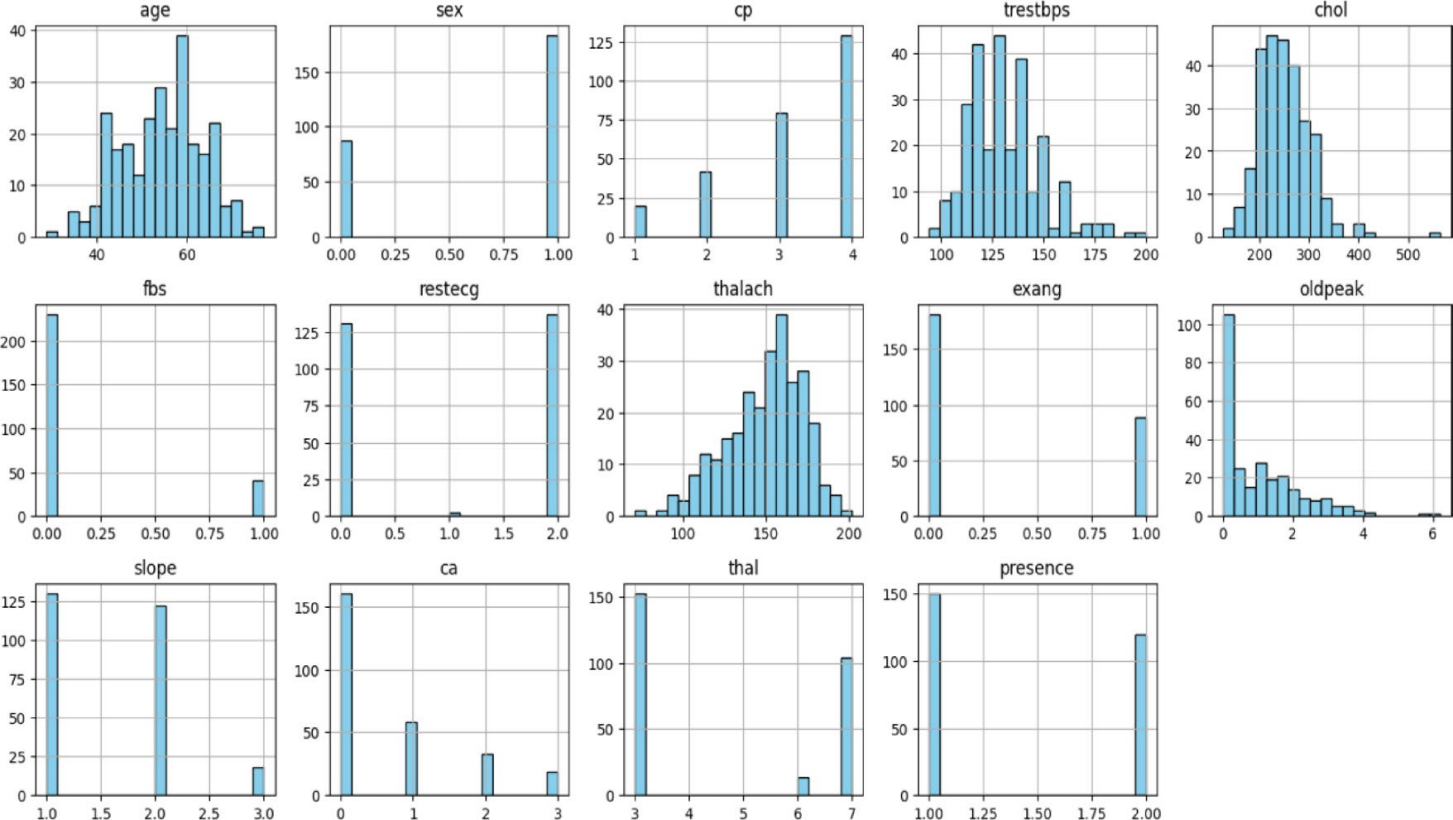
Histogram plot for each feature of Statlog Heart Disease dataset.

**Fig. 5:**
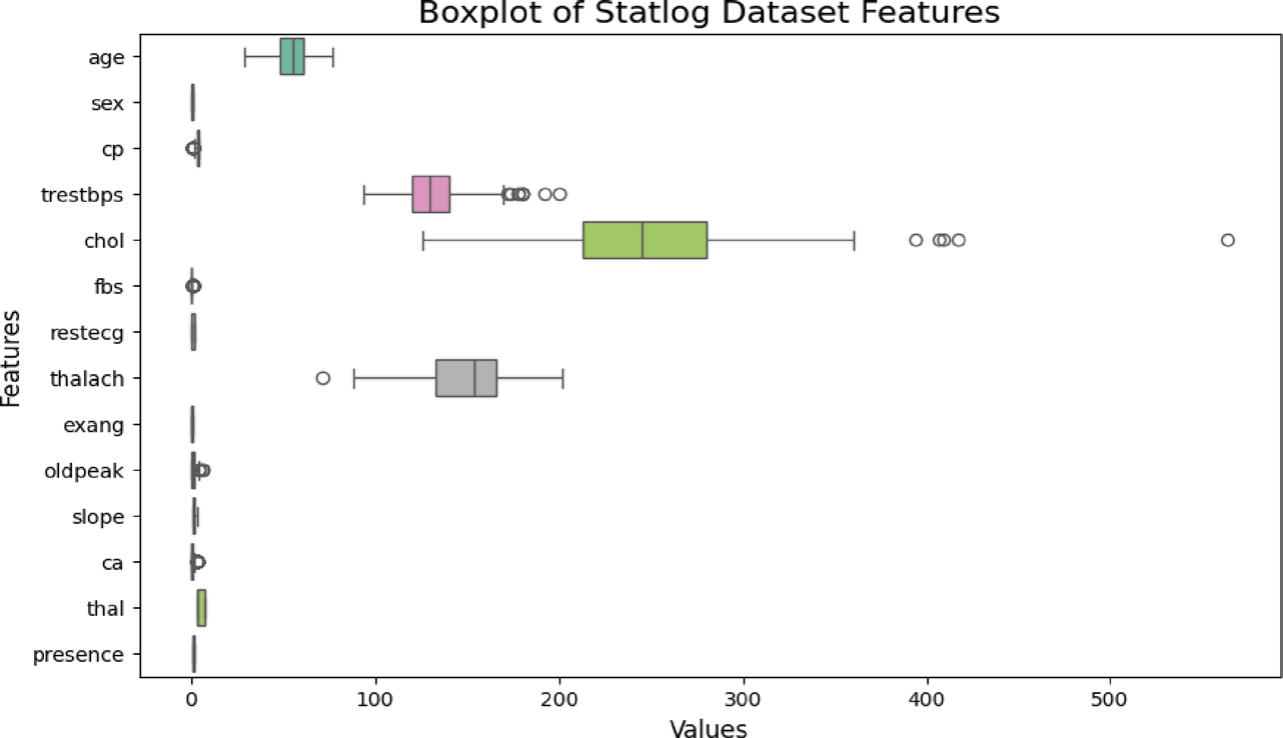
Box plot for each feature of Statlog Heart Disease dataset.

From Tables 1 and 2, it is evident that the extracted datasets contain no missing values and no categorical variables. After checking the dataset, we proceeded with outlier detection. Outliers, which are values that significantly differ from the majority of the data, can impact ML models by causing overfitting, underfitting, or introducing bias. Therefore, identifying and handling outliers is essential for improving model Acc. A common method for detecting outliers is through box plots. These plots visually represent the distribution of data and clearly show potential outliers. By analyzing the box plots, we could identify outliers in the dataset and determine whether they should be removed or handled differently. Figures 3, 4 and 5 present the box plots for all features in the Cleveland and Statlog datasets, respectively.

Histograms were used for each feature in the datasets univariate analysis. This method was chosen because it eliminated the need for the frequently drawn-out categorical encoding that is typically done during the feature engineering stage by representing all of the datasets features as numerical values. In classification machine learning tasks tools like box plots clustered bar plots and histograms are effective for exploratory data analysis (EDA). They help visualize how individual features are distributed and provide information about their variability shape and central tendency. Specifically, by splitting the data range into bins and showing the frequency of observations within each bin histograms offer a simple visual representation of the distribution of a single feature. This provides a general grasp of the data distribution and makes it easier to identify outliers.

Continuous features (such as age blood pressure and cholesterol) that frequently show skewness or different ranges are included in both the Cleveland and Statlog datasets. While some variables like blood pressure age and cholesterol levels can naturally fluctuate extreme values or outliers may indicate errors or unusual events. Since certain variables (like cholesterol) might not follow a normal distribution in medical datasets the IQR method is robust against skewed data. The IQR method uses the median and quartiles, making it more resilient to skewed data or extreme values compared to Z-scores, which assume a normal distribution. IQR provides a clear range for identifying outliers based on the middle 50% of the data (between Q1 and Q3). Outliers are defined as those beyond 1.5 times the IQR from the quartiles, which is simple and interpretable in a medical context. The Cleveland dataset is known to have features with non-normal distributions (for example, cholesterol levels are often positively skewed). In such cases, Z-scores might not be effective because they assume data is normally distributed. IQR, being based on percentiles, doesn’t rely on any specific distribution. However, the Z-score method assumes that the data follow a normal distribution. However, many features in both datasets, like cholesterol, age, and blood pressure, don’t perfectly follow a normal distribution. The histogram plots for all features of both datasets are given in Figs. 2 and 4. Using these plots, distribution of datasets can be shown. The Z-score method is sensitive to extreme values, especially if the data is skewed, leading to more false positives or false negatives when identifying outliers. Hence both heart disease dataset contains both skewed and continuous features, IQR is generally more effective for detecting and removing outliers in this case. It is less sensitive to non-normality in the data and offers a more reliable way of handling outliers compared to the Z-score method. After applying the IQR method to remove outliers and eliminating duplicate rows, the Cleveland dataset was reduced to 229 records with 14 features, including the target variable, while the Statlog dataset was refined to 206 records with 14 features, including the target variable.

Again, as we are using RF and XGB Classifier for classification of datasets, minimal preprocessing (e.g., handling missing values and encoding categorical variables) for RF^72^ and for XGB careful preprocessing (e.g., encoding, handling missing data, and addressing outliers)^73^ is sufficient.

### Performance evaluation measures

The concept of splitting a dataset into subsets for supervised ML classification is a standard practice aimed at evaluating the model’s performance objectively. The dataset is segmented into a training set and a test set, with a validation set potentially included as well. The training set is utilized to create the model, while the test set assesses the model’s ability to generalize to new, unseen data. This separation ensures a trustworthy performance evaluation and helps prevent overfitting. To assess classification performance a confusion matrix (CM) is necessary. By dividing the predictions into True Positives (TP) - correctly identified positive cases—it provides a thorough overview of the classifiers output. True Negatives (TN): Correctly identified negative cases. False Positives (FP): Incorrectly classified positive cases (type I error). False Negatives (FN): Incorrectly classified negative cases (type II error). Through the CM, various metrics can be calculated to assess the performance of a classifier. For example, Acc. (Eq. 3) is determined by the ratio of correctly predicted instances to the total number of predictions, reflecting overall correctness. Rec. or sensitivity (Eq. 4) measures the proportion of actual positive instances correctly identified by the model. Prec. (Eq. 5) evaluates the ratio of correctly predicted positive instances to the total predicted positive instances. Additionally, the F1-S. and AUC (Area Under the Curve) can be computed as shown in Eqs. 6 and 7, respectively, to provide a more comprehensive assessment of the classifier’s performance^74^.

3 $$\:\text{A}\text{c}\text{c}.=\frac{TP+TN}{TP+FP+FN+TN}$$

4 $$\:\text{R}\text{e}\text{c}.=\frac{TP}{TP+FN}$$

5 $$\:\text{P}\text{r}\text{e}\text{c}.=\frac{TP}{TP+FP}$$

6 $$\:\text{F}1-\text{S}.=\frac{2*TP}{2*TP+FP+FN}$$

7 $$\:\text{R}\text{A}\text{U}\text{C}\:\text{s}\text{c}\text{o}\text{r}\text{e}={\int\:}_{x=0}^{1}TPR\left({FPR}^{-1}\left(x\right)\right)dx$$

Where TPR(True Positive Rate) =$\:\frac{TP}{TP+FN}$, FPR(False Positive Rate) =$\:\frac{FP}{FP+TN}$

## Proposed SGO-tuned classifiers

In this study, the primary objective is to optimize the performance of RF and XGB Classifier for predicting HD using the Cleveland and Statlog datasets. We employ SGO algorithm to tune the hyperparameters of these classifiers, aiming to find the optimal set of parameters that maximize predictive Acc. without overfitting or underfitting. The use of SGO ensures a systematic and efficient search of the hyperparameter space, leading to significant performance improvements across all evaluation metrics.

### Approach for hyperparameter tuning of RF and XGB classifier

#### Hyperparameters for RF

RF is an ensemble learning method that operates by constructing multiple decision trees. The performance of the RF model can be significantly influenced by various hyperparameters, which control the behavior of the algorithm. To optimize this study the following hyperparameters need to be set.

- n_estimators: This is the ensembles total number of trees. Although it increases computation time raising this number generally tends to improve model performance.
- max_depth: This is the maximum depth that each tree may have. In addition to preventing overfitting limiting the depth may make it more difficult for the model to identify complex patterns.
- min_samples_split: This is the bare minimum of samples required at an internal node in order to produce division. By limiting the frequency of splits in the tree larger values help to lessen overfitting.
- min_samples_leaf: This parameter specifies how many samples must be present at a leaf node at all times. Particularly when working with noisy datasets it aids in model smoothing.
- max_features: The number of features that are considered when looking for the best split is indicated by the max_features parameter. A lower value reduces the chance of overfitting and encourages more diversity among the trees.
- bootstrap: This parameter indicates if bootstrap samples are used when building a tree. Samples are drawn with replacement when set to True each tree is constructed using the complete dataset when set to False.
- criterion: This specifies the standard by which a splits quality is judged. Options for evaluating information gain include entropy and gini for Gini impurity.
- max_samples: This indicates the maximum quantity of samples that should be chosen from the dataset in order to train each individual estimator. By limiting the size of the training dataset for each tree this parameter helps manage the complexity of the individual trees.

####  Hyperparameters for XGB classifier

A variant of the Gradient Boosting Machine (GBM) algorithm XGB Classifier builds models sequentially with each new model attempting to correct the errors of its predecessors. When adjusting the XGB Classifier for best results the following hyperparameters are essential.

- n_estimators: How many boosting rounds (trees) should be executed? Although more estimators lengthen training time they typically improve the model.
- max_depth: The ultimate depth of each tree separately. Reducing the depth lessens model complexity and helps avoid overfitting.
- subsample: The portion of the entire dataset that will be used to train each tree. Stochastic gradient boosting which can enhance generalization occurs when the value is less than 1point 0.
- learning_rate (eta): Regulates each trees contribution separately. Better generalization is typically the result of smaller values but more trees are needed to reach optimal performance.
- Gamma: The smallest amount of loss reduction needed to create a new partition. By penalizing more intricate models this parameter serves as a regularizer and aids in the control of overfitting.
- Colsample_bytree: This parameter helps reduce overfitting by reducing the model’s reliance on particular features by indicating the percentage of features that are randomly selected for building each tree.
- min_child_weight: This is the smallest instance weight (hessian) that a child node must have. An increased value may help to lessen overfitting.
- reg_alpha: This is the weights L2 regularization term. Through the introduction of a penalty for large weights it mitigates overfitting and controls the strength of regularization.
- colsample_bylevel: The percentage of features to be used at each level of a tree is indicated by the sample_bylevel parameter. It acts as an extra regularization mechanism that limits the number of features available at each split thereby preventing overfitting.

### SGO-tuned classifier model selection for RF and XGB classifier

The hyperparameter selection of machine learning models like RF and XGB Classifier has a significant impact on their efficacy. The SGO algorithm is used by the proposed SGO-tuned Classifier to optimize both models hyperparameters. Using datasets like Cleveland and Statlog this approach enhances their predictive performance for supervised tasks involving the prediction of heart disease.

#### Hyperparameter range selection strategy

The ranges selected for the hyperparameters of both RF and XGB Classifier models were determined using a multi-pronged approach to ensure both relevance and effectiveness within the domain of HDP. This approach combines insights from prior literature, domain expertise, and empirical testing to define a feasible and efficient search space for the SGO-based optimization.

- **Literature review**.
  The initial bounds for key hyperparameters such as n_estimators, max_depth, and learning_rate were drawn from frequently cited configurations in studies involving ensemble learning and clinical prediction tasks. For instance, values such as n_estimators in the range of 100–1000 and max_depth between 5 and 50 are commonly adopted in cardiovascular risk modeling.
- **Domain expertise**:
  Based on best practices in machine learning, parameters were constrained to avoid overly complex or overly simplistic model behavior. For example, restricting subsample and colsample_bytree between 0.1 and 0.9 helped promote model generalization and reduce overfitting. Similarly, bootstrap (True/False) and criterion (gini/entropy) options were included in the RF model based on their effectiveness in various medical data scenarios.
- **Empirical trial-and-error**:
  Preliminary experiments were conducted to validate the practical bounds of the hyperparameters. We iteratively tested edge-case values across small sample runs to exclude configurations that led to extreme underfitting or overfitting. These empirical insights refined our initial assumptions and ensured that the final hyperparameter ranges were neither excessively broad nor too restrictive.

This carefully curated search space allowed the SGO algorithm to explore a meaningful solution space effectively, thus ensuring convergence toward optimal or near-optimal configurations.

**SGO-tuned RF classifier**.

The hyperparameters of the RF model that were optimized in this research consist of:

- n_estimators: Total number of trees (range: 10–1000).
- max_depth: Highest depth allowed for each tree (range: 5–50).
- min_samples_split: Minimum number of samples needed to divide an internal node (range: 2–10).
- min_samples_leaf: the bare minimum of samples (range: 1–number of features) that must be present at a leaf node.
- max_features: Highest number of features considered for making a split (range: 1–number of features).
- bootstrap: Indicates whether bootstrap samples are utilized (True/False).
- criterion: Criterion for splitting (gini/entropy).
- max_samples: Proportion of samples used for constructing each tree (range: 0.25–0.75).

The SGO algorithm translates these hyperparameters into a decision vector consisting of 8 dimensions. The optimization procedure includes decoding the decision vector to set up a RF model, training the model, assessing its performance through Acc., and progressively improving the hyperparameters.

**SGO-tuned XGBClassifier**.

The XGBClassifier model hyperparameters optimized in this study include:

- n_estimators: Number of boosting rounds (range: 50–500).
- max_depth: Maximum depth of trees (range: 3–15).
- subsample: Fraction of samples used per tree (range: 0.1–0.9).
- learning_rate: Contribution of each tree (range: 0.0001–0.2).
- gamma: Minimum loss reduction required to split a node (range: 0–10).
- colsample_bytree: Fraction of features used per tree (range: 0.1–1.0).
- min_child_weight: Minimum sum of instance weight needed in a child (range: 1–10).
- reg_alpha: L1 regularization term (range: 0.001–10).
- colsample_bylevel: Fraction of features used per level (range: 0.1–1.0).

The hyperparameters are encoded into a 9-dimensional decision vector. Like RF, the SGO algorithm optimizes these parameters by iteratively refining the decision vectors based on the fitness value (Acc.).

**Algorithm 2:**
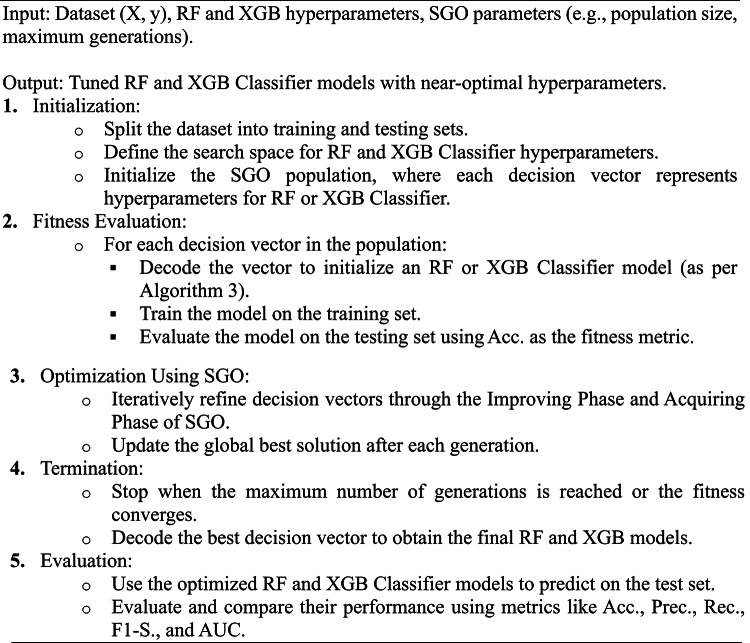
General framework for SGO-tuned classifier.

**Fig. 6:**
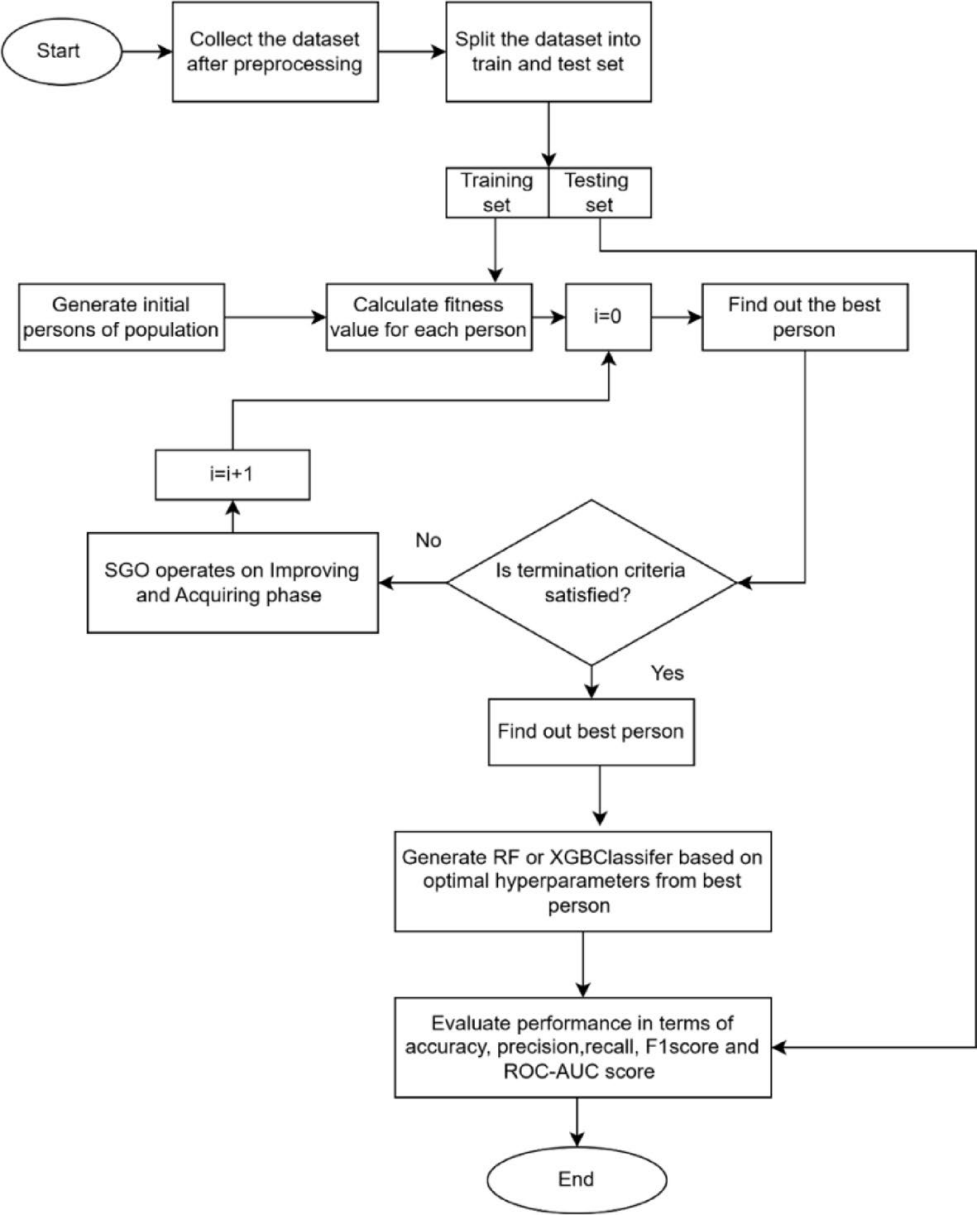
SGO-tuned Classifier model of RF and XGB Classifier.

The flow chart for SGO-tuned Classifier model of RF and XGB Classifier is given in Fig. 6.

**Algorithm 3:**
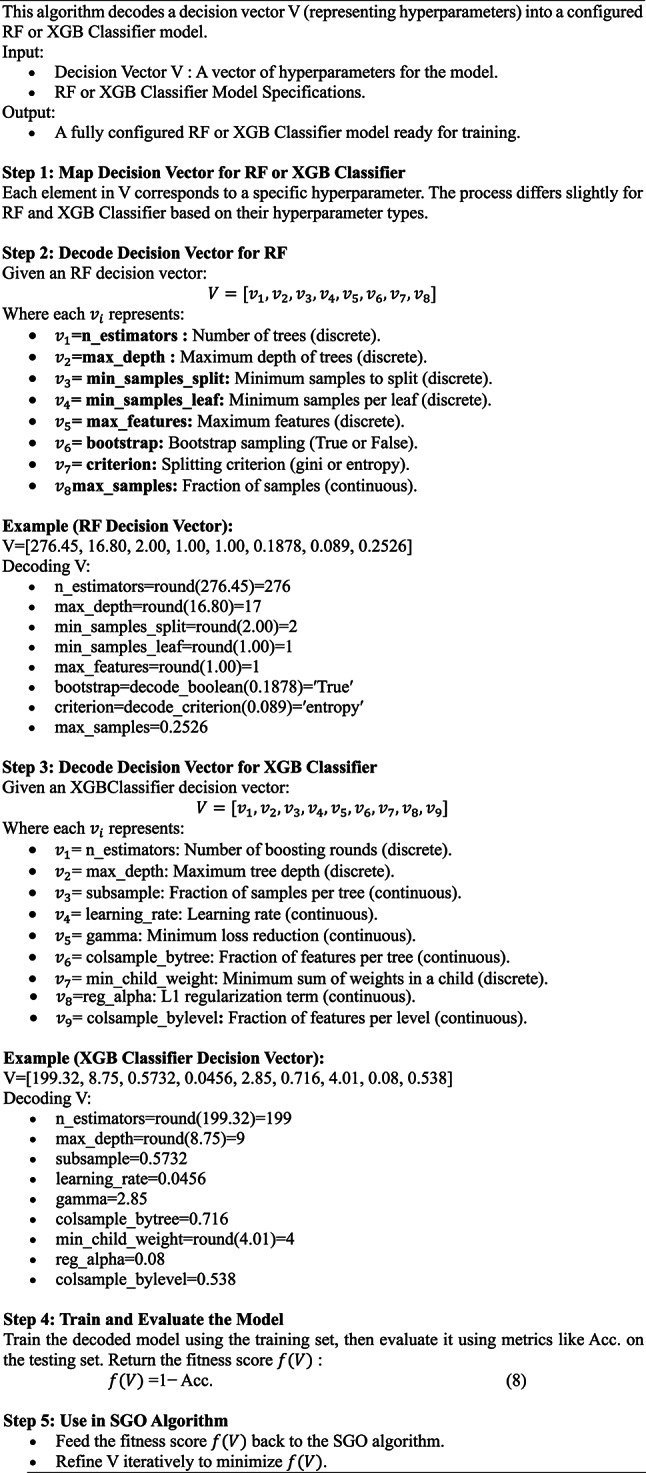
General framework for SGO-tuned classifier.

The SGO algorithm converges by iteratively improving the global best decision vector. At the end of optimization: the RF and XGB Classifier models will have their hyperparameters tuned to achieve the best predictive performance on the given dataset. The optimized models are evaluated and compared to demonstrate the efficacy of the SGO algorithm in improving classification Acc. for heart disease prediction.

**Benefits of the SGO-tuned classifier**.

- Random Forest and XGB Classifier are powerful ensemble models, but their performance depends heavily on hyperparameter tuning.
- The SGO algorithm efficiently navigates the hyperparameter search space to find near-optimal configurations.
- The unified framework allows for a direct comparison of the tuned RF and XGB Classifier models, displaying their respective strengths on the Cleveland and Statlog datasets.

This combined approach ensures comprehensive optimization and provides insights into the relative performance of RF and XGB Classifier when tuned using SGO.

## Experimental design and results analysis

### ML library

The experiments on the ML techniques discussed in this study were conducted using Python, and the Scikit-learn library, commonly known as sklearn, which is a free package for ML. This study also utilized various scientific computing libraries, including Scikit-learn, NumPy^75^, matplotlib, pandas^76^, and seaborn^77^, to support the analysis and implementation.

### Software and tools used

To ensure reproducibility, the entire experimental workflow—ranging from data preprocessing to model training and performance evaluation—was implemented using Python programming language. The following libraries and tools were used:

- Scikit-learn (v1.x) – for implementing RF, model evaluation metrics (Acc., Prec., Rec., F1-s., and CM).
- XGBoost (v1.x) – for implementing the XGB Classifier model.
- NumPy (v1.x) and Pandas (v1.x) – for numerical operations and data manipulation.
- Matplotlib (v3.x) and Seaborn (v0.x) – for data visualization.
- Custom Python implementation of the SGO algorithm – for hyperparameter tuning of both classifiers.

This work was performed using the Standard Google Colab provided by Google as a cloud-based Integrated Development Environment (IDE), with hardware resources an Intel Xeon(R) CPU @ 2.20 GHz with 2 vCPUs (2 virtual CPUs) and 13 GB RAM.

### Experimental setup and repetitions

This experiment presents a comprehensive evaluation of various ML methods, including traditional, optimized, and ensemble-based approaches, along with the proposed SGO-tuned RF and SGO-tuned XGB Classifier methods. To ensure reliable and consistent results, the proposed SGO-tuned RF and SGO-tuned XGB Classifier models were executed 10 times with a stopping criterion of max_FEs = 2000. This repetition accounts for the stochastic nature of metaheuristic algorithms and helps assess their robustness and stability. The performance is evaluated using metrics such as Acc., Prec., Rec., F1-S., RAUC score, CM, and both macro and weighted averages of all metrics. For other methods, the results were imported from their respective prior studies.

### Details of database before classification

Before and after completing the preprocessing step, the datasets were split into two key subsets: 80% of the data was allocated for training, while the remaining 20% was reserved for testing. This division is a crucial step before applying any classification algorithm. Table 5 provides a detailed breakdown of the split datasets. Finally, performance metrics were implemented to assess the prediction outcomes effectively.

**Table 5:** Details of datasets before classification.

Dataset	Total samples (100%)	Training data(80%)			Testing data(20%)		
		Healthy (0)	Sick(1)	Total	Healthy(0)	Sick(1)	Total
Cleveland	303	109	133	242	29	32	61
Statlog	270	99	117	216	33	21	54
Cleveland after preprocessing	229	75	108	183	21	25	46
Statlog after preprocessing	206	100	64	164	22	20	42

### Simulation results: performance comparisons on prediction for both heart disease datasets

#### Preprocessing impact analysis before using SGO-tuned ML

To evaluate the impact of preprocessing on the performance of ML models prior to applying the SGO evolutionary algorithm for hyperparameter tuning, we analyzed the RF and XGB5Classifier on both the Cleveland and Statlog datasets. The comparison was conducted to assess their performance before and after preprocessing, focusing on key metrics such as Acc., Prec., Rec., F1-S., RAUC, macro-averaged metrics, weighted-averaged metrics, and CM components. The results of this evaluation are presented in Table 6.

**Table 6:** Performance evaluation of RF and XGB classifier before and after preprocessing on Cleveland and Statlog DATASETS.

Metrices	Cleveland dataset				Statlog dataset			
	RF	RF with processing	XGB Classifier	XGB Classifier with processing	RF	RF with processing	XGB Classifier	XGB Classifier with processing
Acc.	0.84	0.7826	0.8197	0.8043	0.7963	0.8809	0.8148	0.9286
Prec.	0.84	0.74	0.86	0.77	0.78	0.89	0.79	0.95
Rec.	0.84	0.92	0.78	0.92	0.67	0.85	0.71	0.90
F1-S.	0.84	0.82	0.82	0.84	0.72	0.87	0.75	0.92
RAUC score	0.9203	0.9219	0.9073	0.9238	0.8853	0.9750	0.8846	0.9614
M-Prec.	0.84	0.80	0.82	0.82	0.79	0.88	0.81	0.93
M-Rec.	0.84	0.77	0.82	0.79	0.77	0.88	0.80	0.93
M-F1-S.	0.84	0.77	0.82	0.80	0.78	0.88	0.80	0.93
W-Prec.	0.84	0.80	0.82	0.82	0.79	0.88	0.81	0.93
W-Rec.	0.84	0.78	0.82	0.80	0.80	0.88	0.81	0.93
W-F1-S.	0.84	0.78	0.82	0.80	0.79	0.88	0.81	0.93
TN	24	13	25	14	29	20	29	21
FP	5	8	4	7	4	2	4	1
FN	5	2	7	2	7	3	6	2
TP	27	23	25	23	14	17	15	18

##### Cleveland dataset

For the RF model, Acc. experienced a slight decline from 84 to 78.26% after preprocessing, suggesting the possibility of losing valuable data patterns during the preprocessing stage. Prec. dropped from 84 to 74%, while Rec. saw a notable increase from 84 to 92%, indicating that the model became more effective at identifying true positives but at the cost of a higher false positive rate. The F1-S. showed minor fluctuations, with a slight drop from 84 to 82%, indicating a maintained equilibrium between Prec. and Rec. Furthermore, the RAUC score experienced a small increase from 92.03 to 92.19%, signifying a slight improvement in the model’s ability to distinguish between classes.

With regard to XGB Classifier Acc. after preprocessing saw a slight decrease from 81.97 to 80.43%. The Prec. Saw a notable decline going from 86 to 77% while Rec. showed a significant improvement in sensitivity to true positives, rising from 78 to 92%. F1-S. increased from 82 to 84% primarily as a result of the improvement in Rec. As a result of improved classification performance and increased class distinction the RAUC score also improved rising from 90.73 to 92.38%.

##### Statlog dataset

Regarding the RF model the Acc. greatly enhanced as a result of preprocessing rising from 79.63 to 88.09%. Both Prec. showed significant increases. as well as Rec. from 78 to 89% and from 67 to 85% respectively indicating a decrease in false negatives and an improvement in the identification of true positives. F1-S. showed a noteworthy improvement as well increasing from 72 to 87% suggesting that the model’s efficacy has increased overall. Additionally, the RAUC score rose from 88.53 to 97.50% demonstrating the beneficial impact of preprocessing on the model’s capacity for accurate class distinction. For the XGB Classifier preprocessing yielded a significant Acc. rise reaching 92. 86% from 81. 48%. Prec. saw a notable improvement rising from 79 to 95% while Rec. grew to 90% from 71% previously. The F1-S. also saw a significant rise going from 75 to 92% demonstrating a better balance between Prec. and Rec. Further demonstrating the models improved classification performance after preprocessing the RAUC score increased from 88.46 to 96.14%.

##### CM observations

The Cleveland dataset showed that the XGB Classifier improved Rec. by reducing false negatives (FN) while the RF model increased false positives (FP) at the expense of a minor decrease in Prec. Both RF and XGB Classifier significantly increased true positive (TP) counts and decreased false positives (FN) in the Statlog dataset improving overall performance. Significant improvements in all performance metrics were the result of preprocessing which had a particularly beneficial impact on the Statlog dataset. On the other hand, the Cleveland dataset saw a mixed effect with notable gains in Rec. along with RAUC score but slight declines in Acc. along with Prec. On the Statlog dataset preprocessing had a greater positive impact on the RF model while the XGB Classifier consistently improved across both datasets. After preprocessing RAUC scores significantly increased demonstrating the crucial role preprocessing plays in enhancing the model’s robustness and dependability. All things considered preprocessing helps the models perform better on unseen data and generalize better especially when paired with sophisticated algorithms like RF and XGB Classifier.

#### Performance comparison after using SGO-tuned ML on Cleveland dataset

**Fig. 7:**
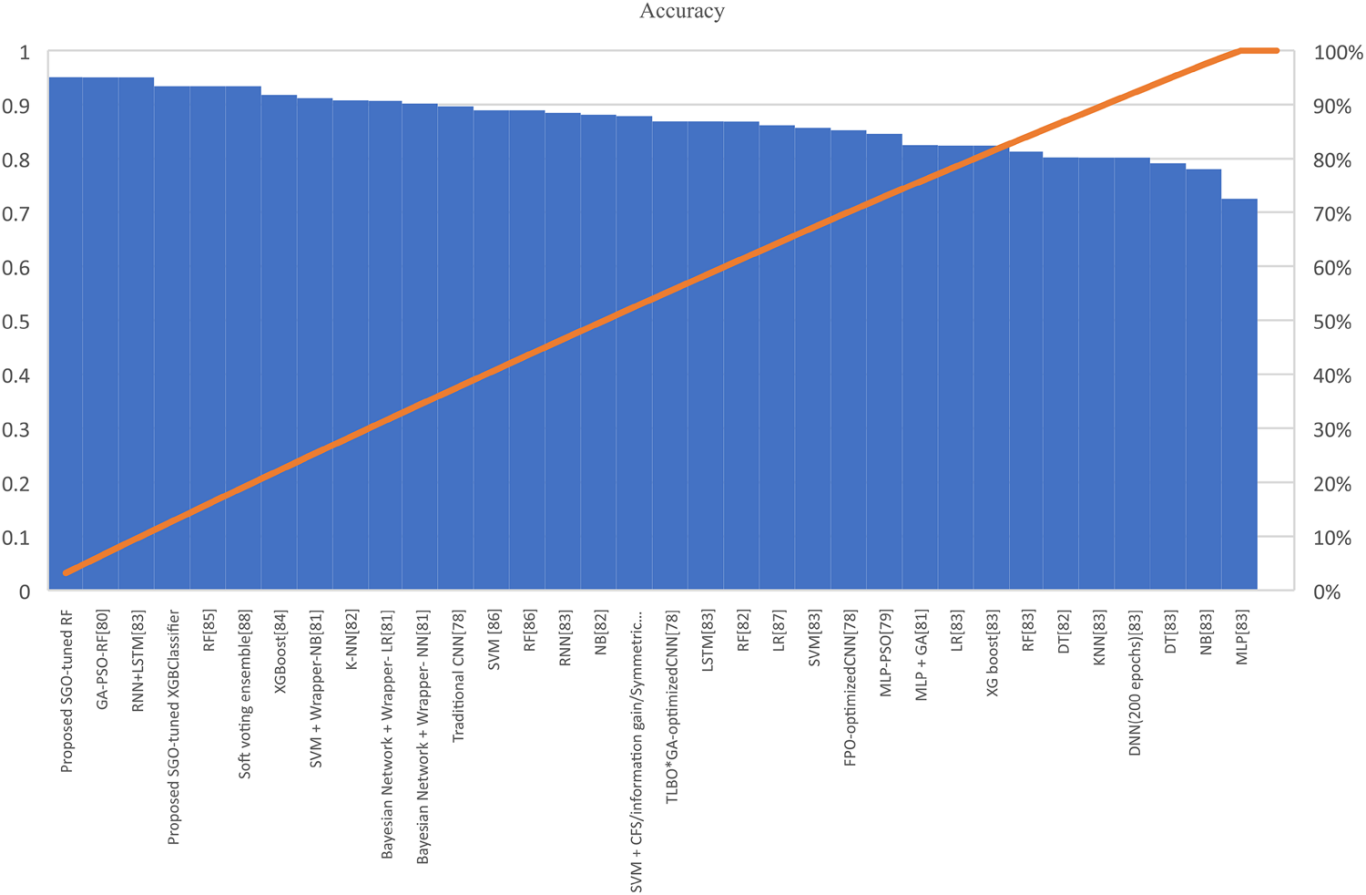
Visual representation of accuracies of all compared algorithms on Cleveland dataset.

**Fig. 8:**
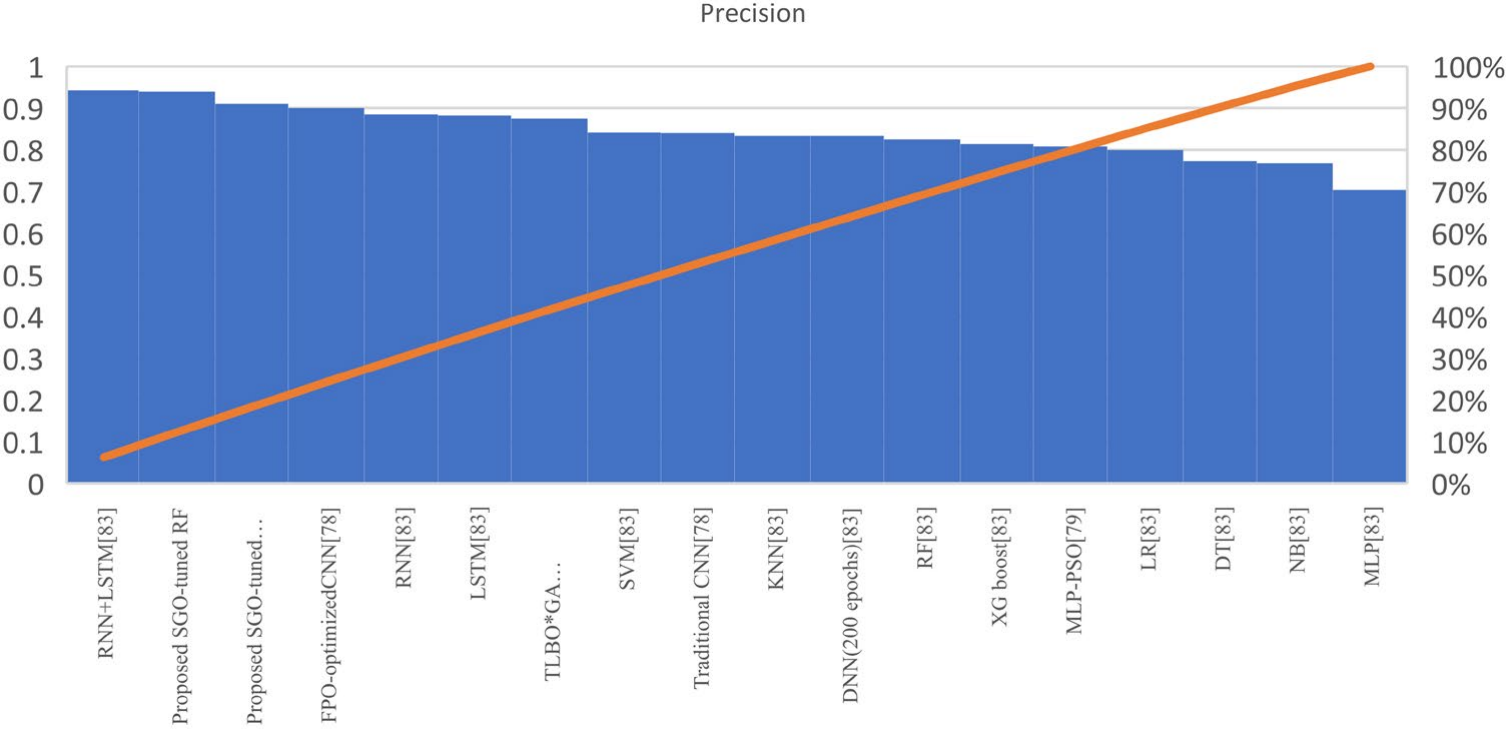
Visual representation of Prec. of all compared algorithms on Cleveland dataset.

**Fig. 9:**
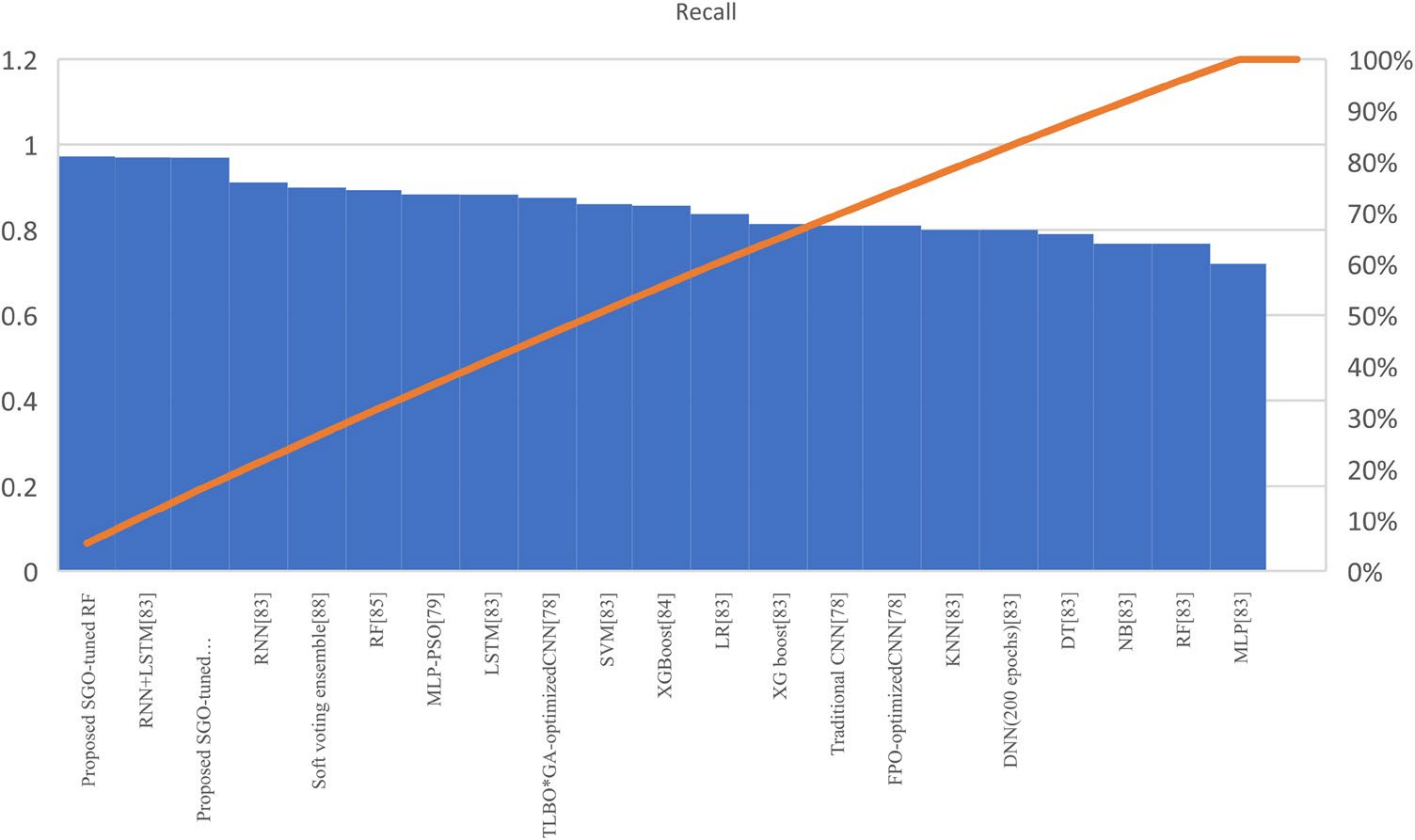
Visual representation of Rec. of all compared algorithms on Cleveland dataset.

**Fig. 10:**
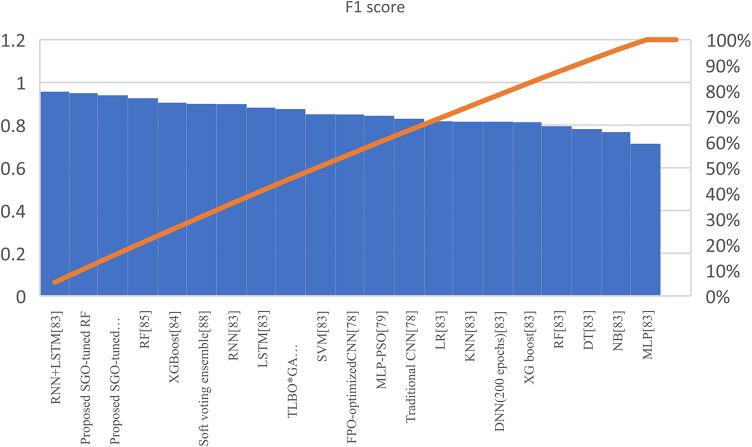
Visual representation of F1-S. of all compared algorithms on Cleveland dataset.

**Fig. 11:**
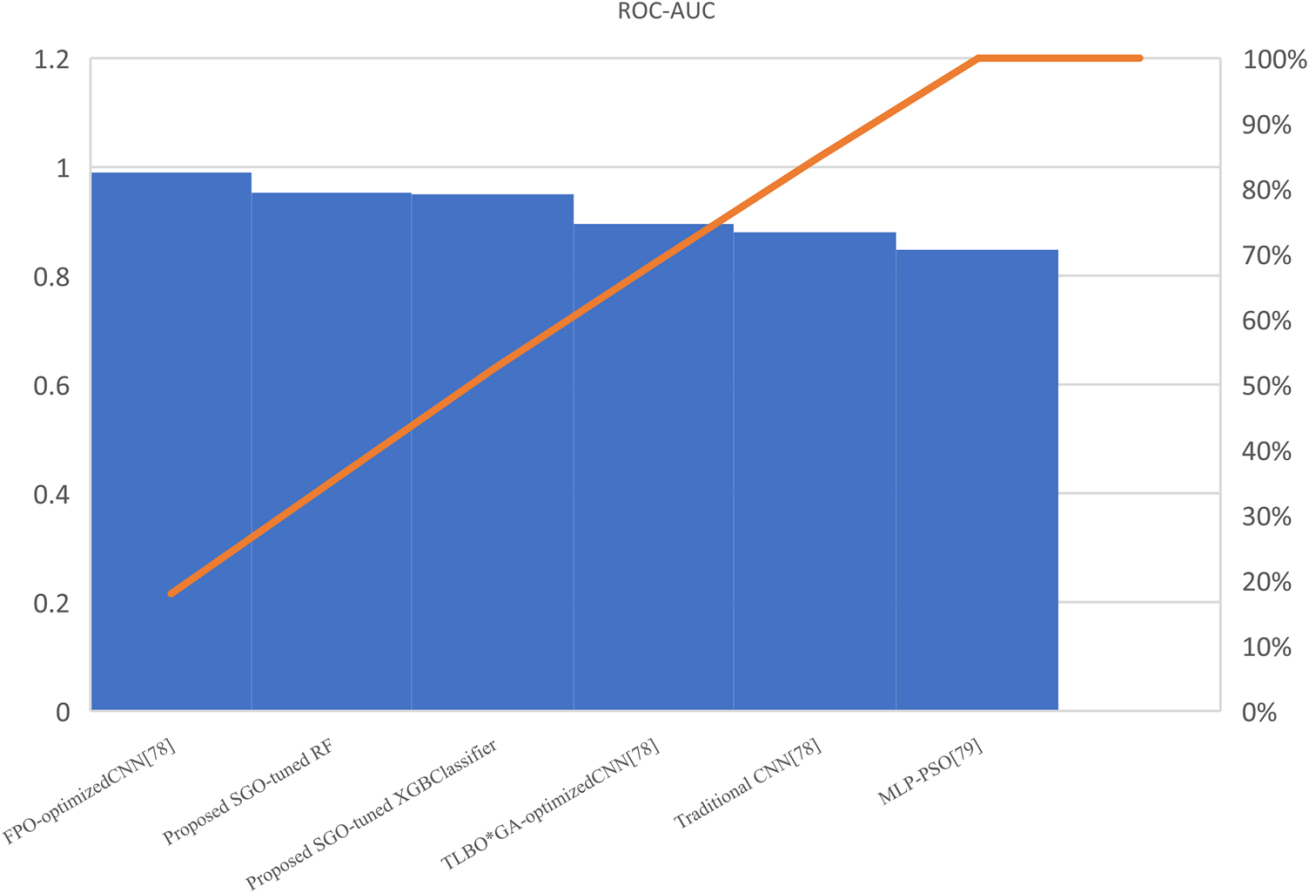
Visual representation on of RAUC score of all compared algorithms on Cleveland dataset.

**Fig. 12:**
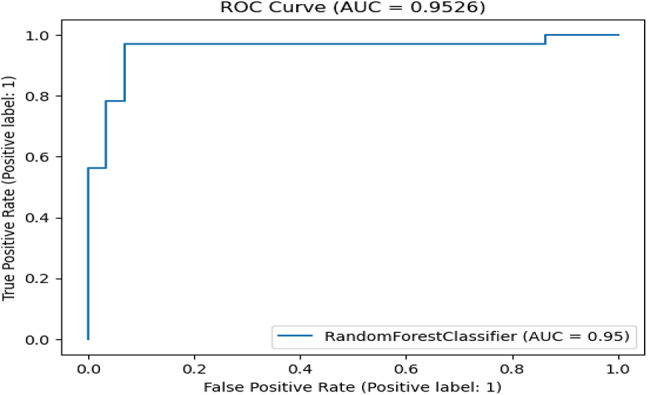
ROC curve of RF algorithm on Cleveland dataset.

**Fig. 13:**
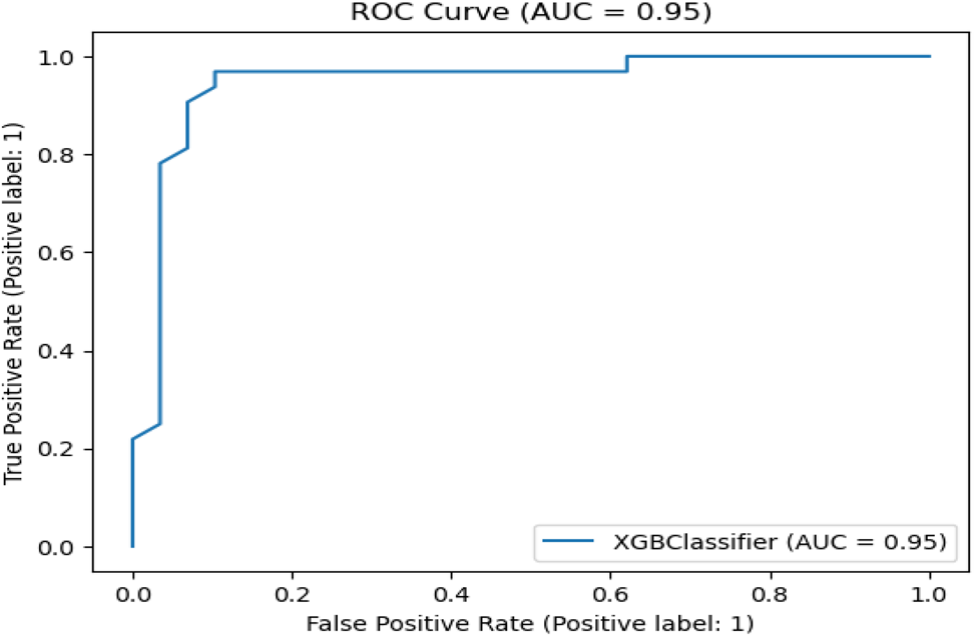
ROC curve of XGB Classifier algorithm on Cleveland dataset.

Table 7 presents the Acc., Prec., Rec., M-F1., and RAUC values, while Table 8 provides the M-Prec., M-Rec., M-F1-S, W-Prec., W-Rec., W-F1-S., and the CM achieved by the proposed SGO-tuned RF, SGO-tuned XGB Classifier, and various peer methods on the Cleveland dataset. Figures 7, 8, 9, 10 and 11 provides a visual performance analysis of these methods in terms of Acc., Prec., Rec., F1-S. and RAUC score respectively. Figures 12 and 13 provide ROC curve of RF algorithm and XGB Classifier algorithm respectively on Cleveland dataset.

**Table 7:** Comparison of various methods with the proposed methods on Cleveland dataset.

Algorithms	Acc.	Prec.	Rec.	F1-S.	RAUC score
Proposed SGO-tuned RF	0.9508	0.94	0.9726	0.95	0.9526
Proposed SGO-tuned XGB Classifier	0.9344	0.91	0.97	0.94	0.95
Traditional CNN^78^	0.897	0.84	0.81	0.83	0.88
FPO-optimizedCNN^78^	0.8525	0.90	0.81	0.85	0.99
TLBO*GA-optimizedCNN^78^	0.869	0.875	0.875	0.875	0.8954
MLP-PSO^79^	0.846	0.808	0.883	0.844	0.848
GA-PSO-RF^80^	0.9506	$\:-$	$\:-$	$\:-$	$\:-$
MLP + GA^61^	0.825	$\:-$	$\:-$	$\:-$	$\:-$
SVM + Wrapper-NB^61^	0.912	$\:-$	$\:-$	$\:-$	$\:-$
SVM + CFS/information gain/Symmetrical uncertainty^61^	0.879	$\:-$	$\:-$	$\:-$	$\:-$
Bayesian Network + Wrapper- LR^61^	0.907	$\:-$	$\:-$	$\:-$	$\:-$
Bayesian Network + Wrapper- NN^61^	0.902	$\:-$	$\:-$	$\:-$	$\:-$
NB^81^	0.8816	$\:-$	$\:-$	$\:-$	$\:-$
K-NN^81^	0.9079	$\:-$	$\:-$	$\:-$	$\:-$
DT^81^	0.8026	$\:-$	$\:-$	$\:-$	$\:-$
RF^81^	0.8684	$\:-$	$\:-$	$\:-$	$\:-$
SVM^82^	0.8571	0.8409	0.8604	0.8505	$\:-$
NB^82^	0.7802	0.7674	0.7674	0.7674	$\:-$
DT^82^	0.7912	0.7727	0.7906	0.7816	$\:-$
RF^82^	0.8131	0.825	0.7674	0.7951	$\:-$
LR^82^	0.8241	0.80	0.8372	0.8181	$\:-$
KNN^82^	0.8021	0.8333	0.80	0.8163	$\:-$
XG boost^82^	0.8241	0.8139	0.8139	0.8139	$\:-$
MLP^82^	0.7252	0.7045	0.7209	0.7126	$\:-$
DNN(200 epochs)^82^	0.8021	0.8333	0.80	0.8163	$\:-$
RNN^82^	0.8852	0.8851	0.9117	0.8985	$\:-$
LSTM^82^	0.8688	0.8823	0.8823	0.8823	$\:-$
RNN + LSTM^82^	0.9505	0.9428	0.9705	0.9565	$\:-$
XGBoost^83^	0.918	$\:-$	0.8571	0.9056	$\:-$
RF^84^	0.9344	$\:-$	0.8928	0.9259	$\:-$
SVM^85^	0.8898	$\:-$	$\:-$	$\:-$	$\:-$
RF^85^	0.8898	$\:-$	$\:-$	$\:-$	$\:-$
LR^86^	0.8617	$\:-$	$\:-$	$\:-$	$\:-$
Soft voting ensemble^87^	0.9344	$\:-$	0.90	0.90	$\:-$

**Table 8:** Comparison of various methods with the proposed methods on Cleveland dataset.

Algorithms	Proposed SGO-tuned RF	Proposed SGO-tuned XGB Classifier	Traditional CNN[50]	TLBO*GA-optimized CNN [50]
M-Prec.	0.95	0.94	$\:-$	$\:-$
M-Rec.	0.95	0.93	$\:-$	$\:-$
M-F1-S.	0.95	0.93	$\:-$	$\:-$
W-Prec.	0.95	0.94	$\:-$	$\:-$
W-Rec.	0.95	0.93	$\:-$	$\:-$
W-F1-S.	0.95	0.93	$\:-$	$\:-$
Ture negative	27	26	25	25
False positive	2	3	4	4
True positive	31	31	25	28
False negative	1	1	7	4

## Discussion and analysis of results

The comparison Tables 7 and 8 present a comprehensive evaluation of various ML methods, including traditional, optimized, and ensemble-based approaches, alongside the proposed SGO-tuned RF and SGO-tuned XGB Classifier methods. The results are analyzed based on evaluation metrics such as Acc., Prec., Rec., F1-S., RAUC score, CM, macro and weighted average of all metrices with references included to acknowledge prior studies.

### Overall performance

The proposed SGO-tuned RF achieves the highest Acc. (0.9508) and RAUC score (0.9526) among most methods, indicating superior performance in correctly identifying heart disease cases while maintaining a strong balance between true positives and false positives. High Prec. (0.94) suggests the model’s ability to minimize false positives. Rec. (0.97) emphasizes the model’s strength in capturing true positive cases. The proposed SGO-tuned XGB Classifier delivers robust performance with an Acc. of 0.9344 and a high RAUC score of 0.95, showcasing its effectiveness in handling the classification task. The model demonstrates excellent Rec. (0.97), which is crucial for detecting critical cases, though its Prec. (0.91) is slightly lower than SGO-tuned RF, indicating slightly more false positives.

The conventional CNN demonstrates moderate performance, reaching an Acc. of just 81.97% and an RAUC score of 88, which underscores its challenges in identifying intricate patterns within the dataset^78^. The FPO-optimized CNN and TLBO + GA-optimized CNN improve over the traditional CNN with accuracies of 85.25% and 86.9%, respectively. However, these methods still fall short compared to the proposed SGO-tuned models in terms of Acc. and F1-S^78,79^.

The RF model, without SGO optimization, achieves accuracies ranging from 86.84 to 93.44% in different studies^81,84^. This showcases the inherent strength of RF in handling tabular datasets. However, the proposed SGO-tuned RF significantly improves upon these results, demonstrating the impact of hyperparameter tuning. SVM models achieve accuracies in the range of 83.5–91.2%, depending on the feature selection or wrapper methods applied^61,85^. These results are competitive but still lag behind the proposed SGO-tuned methods in terms of both Acc. and Rec. Models like NB, DT, LR, and KNN achieve accuracies below 90% in most studies^73,82^. While these models are simpler and computationally efficient, their performance does not match the complexity and Acc. of ensemble-based and optimized models. Achieves a high Acc. of 93.44%, showing the power of combining multiple models^87^. However, the Prec. and Rec. values are slightly lower than those of the proposed SGO-tuned RF and XGB Classifier. RNN and LSTM models show promising results, with RNN + LSTM achieving an Acc. of 95.06%^82^. While comparable to the proposed SGO-tuned RF, these models generally require more computational resources and are more complex to train.

The SGO-tuned RF outperforms other models in macro-averaged and weighted metrics, achieving 0.95 across Prec., Rec., and F1-S. SGO-tuned XGB Classifier maintains a close performance with macro-averaged and W-F1-S. of 0.93. The CM reveals a significantly lower number of false positives (2 for SGO-tuned RF vs. 4 for TLBO*GA-optimized CNN) and false negatives (1 vs. 7 for Traditional CNN).

The proposed SGO-tuned RF and XGB Classifier models outperform their non-optimized counterparts and most other methods in terms of Acc. and Rec., emphasizing the importance of hyperparameter optimization in achieving superior results. Both proposed methods maintain a strong balance between Prec. and Rec., critical for heart disease prediction, where minimizing false negatives is paramount. Ensemble-based models such as RF and XGB Classifier demonstrate superior performance due to their ability to handle imbalanced data, noise, and complex patterns effectively.

In overall it can be shown that results highlight the effectiveness of the SGO algorithm in optimizing hyperparameters for RF and XGB Classifier. The proposed SGO-tuned RF emerges as the most accurate and reliable method, while the SGO-tuned XGB Classifier offers competitive performance with slight trade-offs in Prec. Compared to traditional methods, DL approaches, and other optimized techniques, the proposed models provide a significant improvement in Acc., Rec., and overall robustness, making them ideal for heart disease prediction on the Cleveland dataset^61,78,82,85,87^.

### Detailed graphical observation

A bar chart can illustrate the Acc. of different algorithms. This highlights the SGO-tuned RF (0.9508) and SGO-tuned XGBClassifier (0.9344) as having the highest accuracies compared to traditional algorithms such as MLP + GA (0.825) and NB (0.7802).A grouped bar chart for Prec. and Rec. can show that SGO-tuned RF and XGBClassifier excel in balancing these metrics. Notably, the RNN + LSTM achieves comparable performance with high Rec. (0.9705). A line chart for F1-S. reveals that SGO-tuned RF (0.95), RNN + LSTM (0.9565), and SGO-tuned XGBClassifier (0.94) are among the top-performing models. Traditional algorithms like MLP + GA and DT lag with lower F1-S.s. A scatter plot for RAUC scores can demonstrate the superior generalization capability of SGO-tuned RF (0.9526) and SGO-tuned XGBClassifier (0.95) compared to traditional CNN-based models, such as FPO-optimized CNN (0.99 RAUC), but with lower Acc.

The SGO-tuned RF consistently achieves the highest scores across metrics, making it a robust and reliable choice for prediction systems. The SGO-tuned XGBClassifier closely follows, showing its effectiveness in scenarios requiring high Rec. and generalization. The RNN + LSTM is highly competitive, excelling in Rec. (0.9705) and F1-S. (0.9565), comparable to the proposed SGO methods. Traditional ensemble techniques like soft voting also perform well, though they do not outperform SGO-tuned algorithms. Models such as MLP + GA and Bayesian network-based approaches fall short on several metrics, demonstrating limited effectiveness compared to modern optimization-based techniques. Classical ML models (e.g., NB, SVM) have lower Acc. and do not effectively balance Prec. and Rec. The high RAUC score of the FPO-optimized CNN suggests it has excellent discrimination ability but is less reliable for real-world applications due to lower Acc. and Prec. While some models excel in one metric (e.g., FPO-optimized CNN in RAUC), their performance across other metrics must be considered for practical applications. The proposed SGO-tuned algorithms achieve a consistent balance across metrics, supporting their deployment in real-world scenarios.

**Table 9:** List of hyperparameters for random forest find by SGO using Cleveland dataset.

Sl. no	n_estimators	max_depth	min_samples_split	min_samples_leaf	max_features	Bootstrap	Criterion	max_samples
1	15	5	4	5	1	True	entropy	0.25
2	15	8	3	5	1	True	entropy	0.25
3	15	11	3	5	1	True	entropy	0.25
4	15	6	2	5	1	True	entropy	0.25
5	15	5	2	5	1	True	entropy	0.25
6	15	7	2	5	1	True	entropy	0.25
7	15	14	2	5	1	True	entropy	0.25
8	16	4	3	5	1	True	entropy	0.25
9	15	11	2	5	1	True	entropy	0.25
10	15	5	4	5	1	True	entropy	0.25
11	15	8	3	5	1	True	entropy	0.25
12	15	8	2	5	1	True	entropy	0.25
13	15	5	3	5	1	True	entropy	0.25
14	15	21	2	5	1	True	entropy	0.25
15	15	18	5	1	1	True	entropy	0.25
16	15	4	2	5	1	True	entropy	0.25
17	15	7	3	5	1	True	entropy	0.25
18	15	15	2	5	1	True	entropy	0.25
19	15	13	2	5	1	True	entropy	0.25
20	15	12	2	5	1	True	entropy	0.25

**Table 10:** List of hyperparameters for XGB classifier find by SGO using Cleveland dataset.

Sl. no	n_estimators	max_depth	Subsample	Learning_rate	Gamma	Cosample_bytree	Min_child_weight	Reg_alpha	Cosample_bylevel
1	244	8	0.36	0.070	6.05	0.56	7.83	3.35	0.57
2	81	7	0.46	0.034	8.07	0.95	9.66	0.9	0.88
3	344	15	0.48	0.2	7.9	6.18	10	2.11	0.50
4	50	14	0.38	0.13	9.34	0.59	7.42	0.37	0.38
5	500	8	0.29	0.11	6.20	0.55	7.86	3.64	0.64
6	410	11	0.37	0.1	4.75	0.53	8.87	5.82	0.64
7	325	4	0.41	0.19	6.65	0.13	9.00	3.90	1.00
8	451	13	0.21	0.15	2.91	0.76	5.91	3.28	0.26
9	140	7	0.22	0.07	1.48	0.24	1.33	4.11	0.44
10	306	12	0.32	0.08	2.60	0.49	2.96	4.23	0.36
11	232	9	0.24	0.19	1.32	0.56	6.22	5.36	0.92
12	500	10	0.34	0.14	1.90	0.86	8.15	6.07	0.49
13	151	7	0.60	0.13	0.00	0.18	1.00	9.08	0.12
14	160	7	0.27	0.16	7.81	1.00	7.09	1.40	0.1
15	374	6	0.48	0.16	1.68	0.60	2.02	6.56	0.81
16	334	8	0.37	0.1	1.82	0.97	8.85	6.17	0.88
17	88	5	0.1	0.16	4.42	0.60	1.78	0.21	0.23
18	50	3	0.28	0.13	2.53	0.1	3.47	4.59	0.69
19	249	6	0.32	0.07	2.71	0.56	7.08	5.31	0.77
20	500	8	0.42	0.12	9.05	0.76	9.88	2.40	0.88

Table 9 presents 20 hyperparameter configurations for RF identified using SGO on the Cleveland dataset. Notably, all these configurations achieved identical performance metrics: Acc. (0.9508), Prec. (0.94), Rec. (0.9726), F1-S. (0.95), and RAUC Score (0.9526), highlighting the robustness and reliability of the optimization process. Similarly, Table 10 lists 20 hyperparameter configurations for XGB Classifier derived through SGO on the Cleveland dataset. All configurations consistently achieved the same performance metrics: Acc. (0.9344), Prec. (0.91), Rec. (0.97), F1-S. (0.94), and RAUC Score (0.95), further emphasizing the effectiveness of the optimization approach.

#### Performance comparison after using SGO-tuned ML on Statlog dataset

**Fig. 14:**
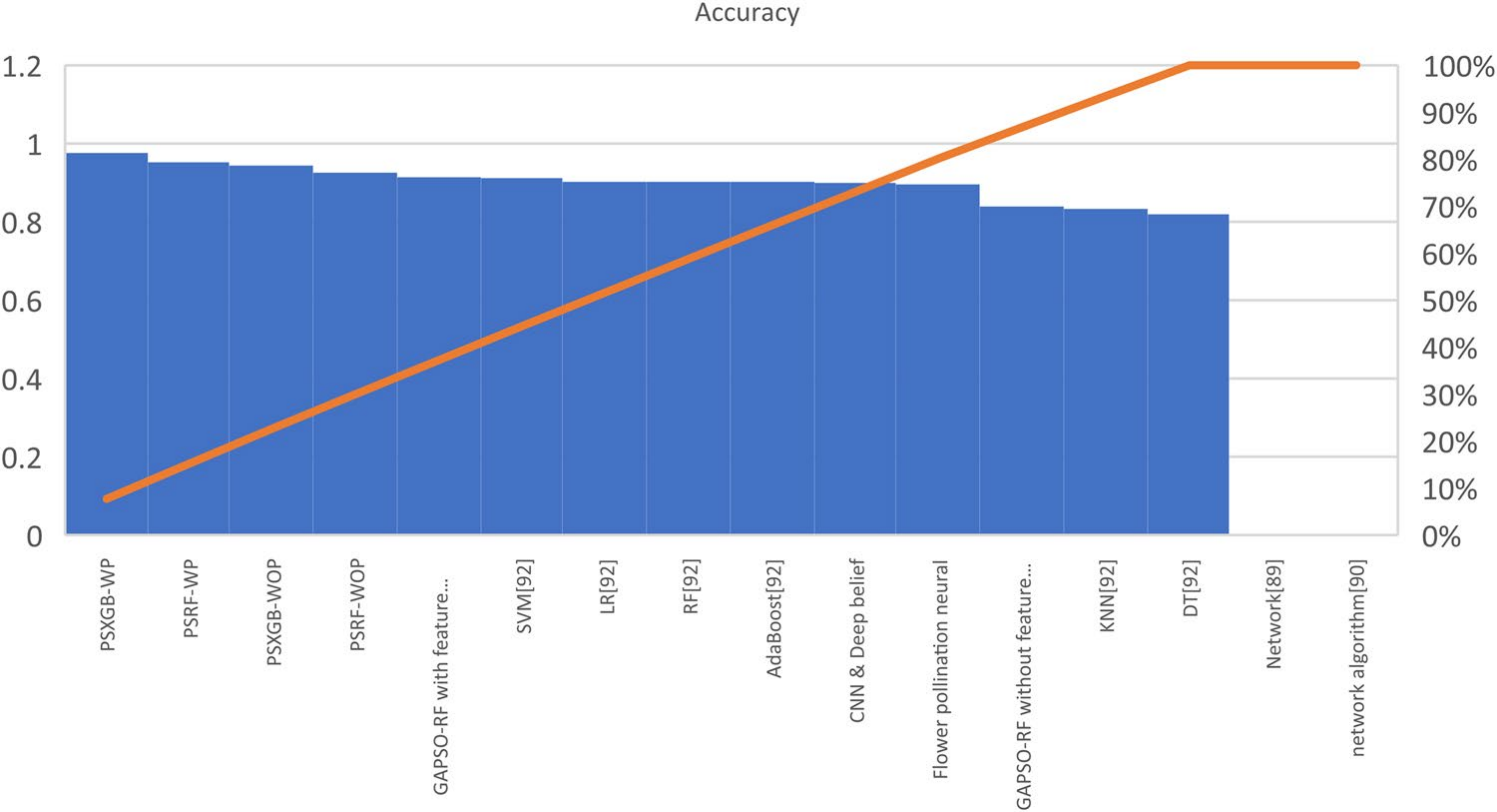
Visual representation of Accuracies of all compared algorithms on Statlog dataset.

**Fig. 15:**
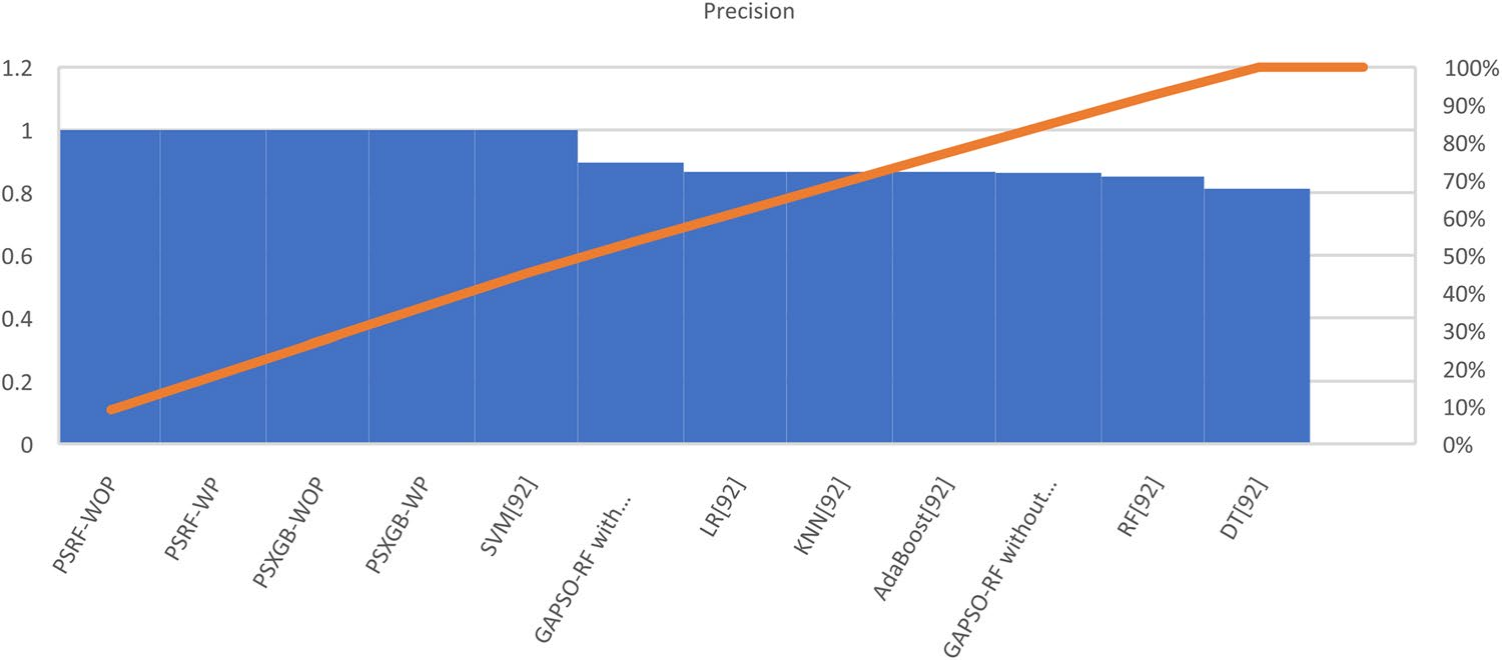
Visual representation of Prec. of all compared algorithms on Statlog dataset.

**Fig. 16:**
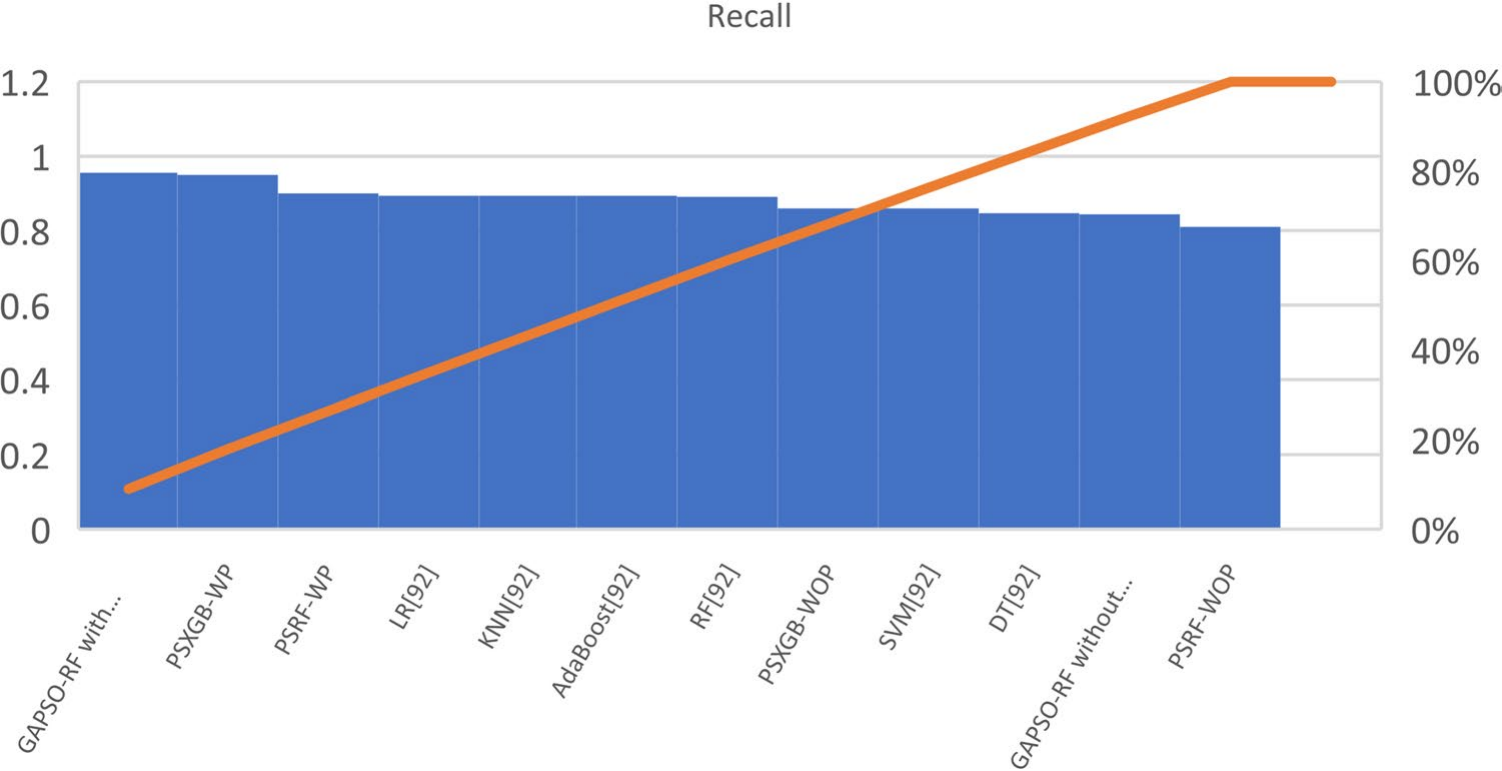
Visual representation of Rec. of all compared algorithms on Statlog dataset.

**Fig. 17:**
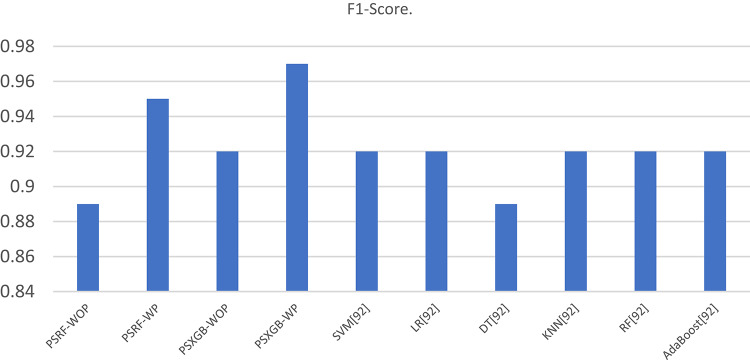
Visual representation of F1-S. of all compared algorithms on Statlog dataset.

**Fig. 18:**
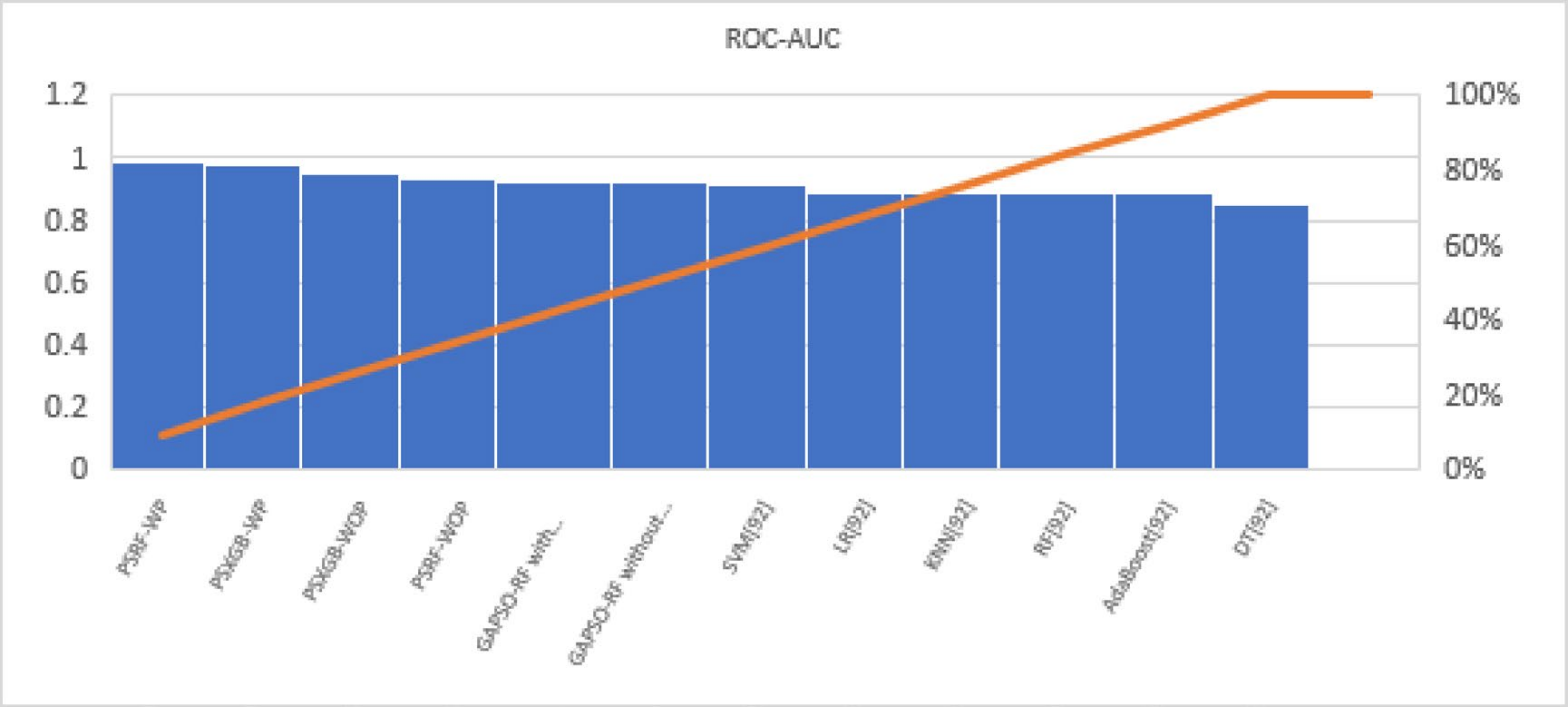
Visual representation of RAUC score of all compared algorithms on Statlog dataset.

**Fig. 19:**
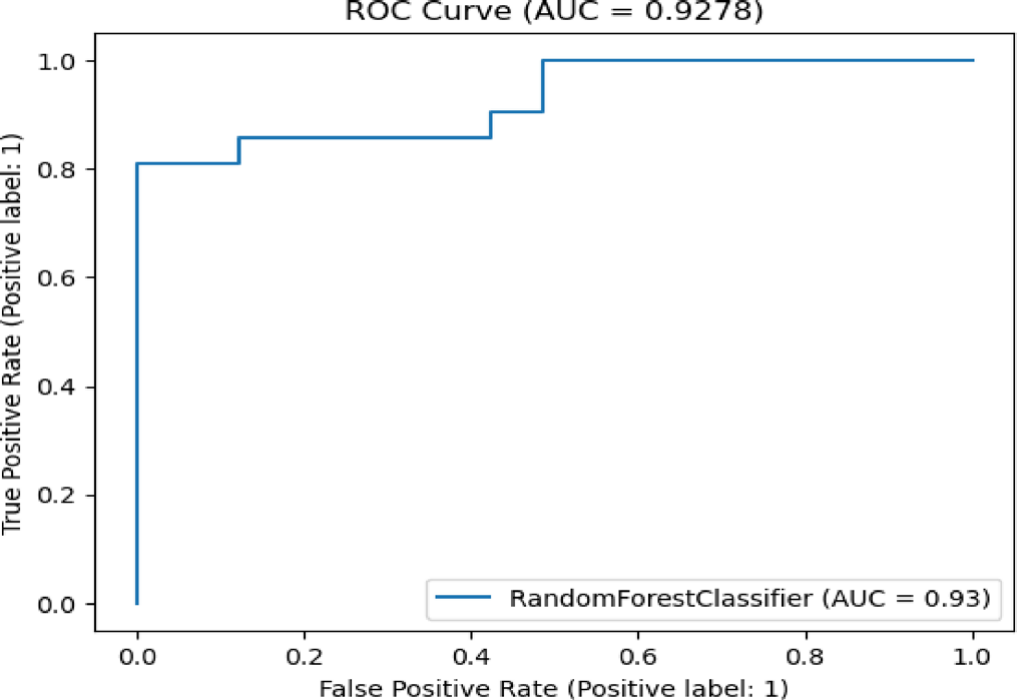
ROC curve of RF algorithm on Statlog dataset without preprocessing.

**Fig. 20:**
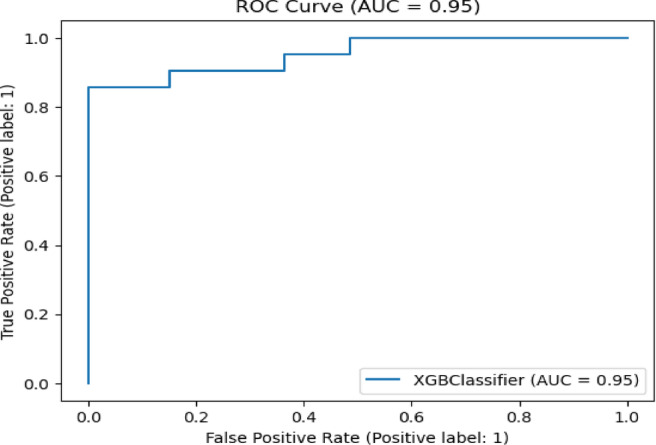
ROC curve of XGB Classifier algorithm on Statlog dataset without preprocessing.

**Fig. 21:**
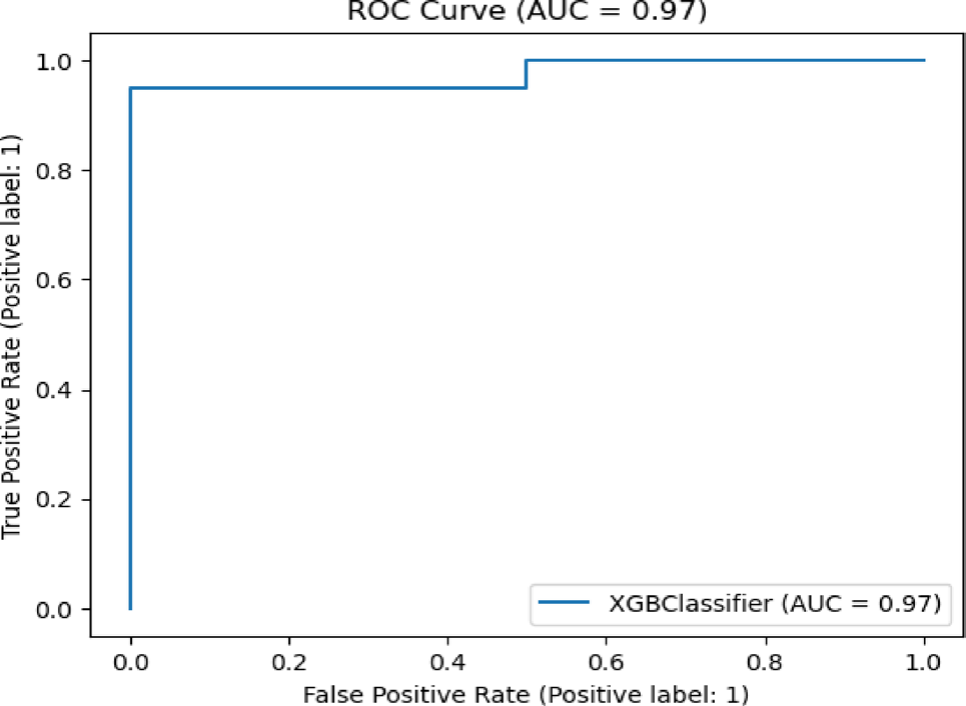
ROC curve of XGB Classifier algorithm on Statlog dataset with preprocessing.

**Fig. 22:**
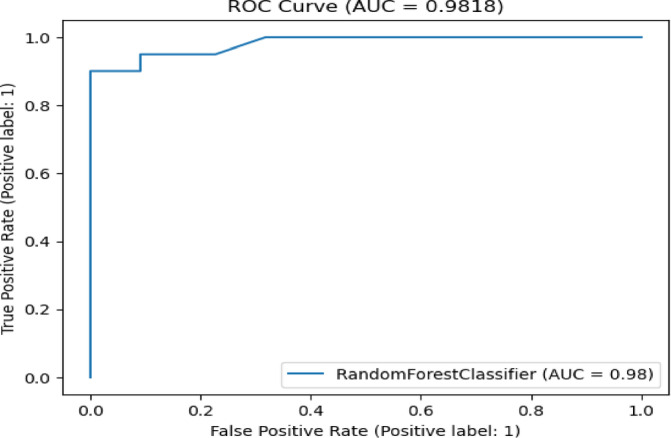
ROC curve of RF algorithm on Statlog dataset with preprocessing.

Table 11 presents the Acc., Prec., Rec., F1-S., and RAUC values, while Table 12 provides the M-Prec., M-Rec., M-F1-S., W-Prec., W-Rec., W-F1-S., and the CM achieved by the proposed SGO-tuned RF, SGO-tuned XGB Classifier, and various peer methods on the Statlog dataset. Figures 14, 15, 16, 17 and 18 provides a visual performance analysis of these methods in terms of Acc., Prec., Rec., F1-S., and RAUC score respectively. Figures 19 and 20 shows the ROC curve of RF algorithm and XGB Classifier algorithm on Statlog dataset without preprocessing respectively. Similarly, Figs. 21 and 22 show ROC curve of XGB Classifier algorithm and RF algorithm on Statlog dataset with preprocessing respectively.

**Table 11:** Comparison of various methods with the proposed methods on Statlog dataset.

Algorithms	Acc.	Prec.	Rec.	F1-S.	RAUC score
PSRF-WOP	0.9259	1.00	0.81	0.89	0.9278
PSRF-WP	0.9524	1.00	0.90	0.95	0.9818
PSXGB-WOP	0.9444	1.00	0.86	0.92	0.9524
PSXGB-WP	0.9762	1.00	0.95	0.97	0.9750
Flower pollination neural network^88^	0.8960	$\:-$	$\:-$	$\:-$	$\:-$
CNN & Deep belief network algorithm^89^	0.9000	$\:-$	$\:-$	$\:-$	$\:-$
GAPSO-RF without feature selection^80^	0.8395	0.8636	0.8444	$\:-$	0.9190
GAPSO-RF with feature selection^80^	0.9140	0.8958	0.9556	$\:-$	0.9260
SVM^90^	0.9118	1.00	0.8600	0.9200	0.9100
LR^90^	0.9028	0.8667	0.8938	0.9200	0.8900
DT^90^	0.8194	0.8125	0.8469	0.8900	0.8500
KNN^90^	0.8333	0.8667	0.8938	0.9200	0.8900
RF^90^	0.9028	0.8511	0.8906	0.9200	0.8900
AdaBoost^90^	0.9028	0.8667	0.8938	0.9200	0.8900

PSRF-WOP: Proposed SGO-tuned RF without preprocessing, PSRF-WP: Proposed SGO-tuned RF with preprocessing, PSXGB-WOP : Proposed SGO-tuned XGBClassifier without preprocessing, PSXGB-WP : Proposed SGO-tuned XGB Classifier with preprocessing.

**Table 12:** Comparison of various methods with the proposed methods on Statlog dataset.

Algorithms	PSRF-WOP	PSRF-WP	PSXGB-WOP	PSXGB-WP	SVM^90^	LR^90^	DT^90^	KNN^90^	AdaBoost^90^
M-Prec.	0.95	0.96	0.96	0.98	0.9348	0.9150	0.8150	0.9150	0.9150
M-Rec.	0.90	0.95	0.93	0.97	0.9100	0.8938	0.7937	0.8937	0.8938
M-F1-S.	0.92	0.95	0.94	0.98	0.9134	0.9000	0.7800	0.9000	0.9000
W-Prec.	0.93	0.96	0.95	0.98	0.9300	0.9100	0.8109	0.9100	0.9100
W-Rec.	0.93	0.95	0.94	0.98	0.92	0.9028	0.8100	0.9027	0.9028
W-F1-S.	0.92	0.95	0.94	0.98	0.92	0.9015	0.8023	0.9015	0.9018
Ture negative	33	22	33	22	$\:-$	$\:-$	$\:-$	$\:-$	$\:-$
False positive	0	0	0	0	$\:-$	$\:-$	$\:-$	$\:-$	$\:-$
True positive	17	18	18	19	$\:-$	$\:-$	$\:-$	$\:-$	$\:-$
False negative	4	2	3	1	$\:-$	$\:-$	$\:-$	$\:-$	$\:-$

## Discussion and analysis of results

Tables 11 and 12 present a detailed comparison of the proposed SGO-tuned RF and SGO-tuned XGB Classifier, with and without preprocessing, alongside various peer methods such as Flower Pollination Neural Network^88^, CNN & Deep Belief Network^89^, GAPSO-RF^80^, SVM^90^, LR^90^, DT^90^, KNN^90^, and AdaBoost^90^. The analysis highlights the impact of preprocessing and the SGO algorithm on model performance for the Statlog dataset.

The SGO-tuned RF and XGB Classifier achieved the highest Acc. among all methods. The SGO-tuned XGB Classifier with preprocessing recorded the best Acc. at 97.62%, followed by the SGO-tuned RF with preprocessing at 95.24%. Peer methods like Flower Pollination Neural Network^61^ and CNN & Deep Belief Network^62^ reported accuracies of 89.60% and 90.00%, respectively, but did not match the performance of the proposed models. Both SGO-tuned RF and XGB Classifier demonstrated 100% Prec. across all configurations, emphasizing their effectiveness in identifying true positives with zero false positives. Preprocessing significantly boosted Rec., with the SGO-tuned RF rising from 81 to 90%, while the SGO-tuned XGB Classifier improved from 86 to 95%. This enhancement illustrates the models’ increased sensitivity following preprocessing. F1-S. significant gains were also observed after preprocessing as the SGO-tuned XGB Classifier obtained the highest F1-S. of 97%, suggesting a solid and well-balanced achievement. Additionally, RAUC scores improved with the SGO-tuned XGB Classifier reaching 97.50% and the SGO-tuned RF reaching 98.18%. These results validate how well the models can distinguish between classes.

Significant improvements in macro-averaged Prec. were shown by the suggested models. Rec. and F1-S. using preprocessing and the SGO-tuned XGB Classifier achieved metrics of 0.98 in all three categories. A similar pattern was also shown by weighted metrics, which supported the model’s dependability across different class distributions. Preprocessing improved Rec. by reducing false negatives from four to two for the SGO-tuned RF. and making sure that no false positives occurred. Likewise, the SGO-tuned XGB Classifiers preprocessing reduced false negatives from three to one, boosting its sensitivity and overall efficacy.

Using feature selection GAPSO-RF^80^ achieved an Acc. of 91 to 40%, demonstrating the benefits of feature selection did not meet the Recs. standards. as well as the suggested SGO-tuned models RAUC score values. LR^90^, AdaBoost^90^, and SVM^90^ performed similarly on some metrics, but they couldn’t achieve the high Acc. or F1-S. of the methods that have been proposed.

The SGO-tuned RF and SGO-tuned XGB Classifier, especially when combined with preprocessing, achieve state-of-the-art performance on the Statlog dataset. The SGO algorithm’s hyperparameter tuning substantially improves the models’ Acc., Prec., Rec., and F1-S., as evidenced by their high RAUC scores. Additionally, preprocessing enhances these benefits by refining data quality and minimizing noise. These results confirm the effectiveness of the proposed approach and its potential for real-world classification applications.

**Table 13:** List of hyperparameters for RF find by SGO using Stalog dataset without preprocessing.

Sl. no	n_estimators	max_depth	min_samples_split	min_samples_leaf	max_features	bootstrap	Criterion	max_samples
1	173	50	5	1	2	True	entropy	0.3061
2	56	7	2	1	2	True	entropy	0.2574

**Table 14:** List of hyperparameters for XGB classifier find by SGO using Stalog dataset without preprocessing.

Sl. no	n_estimators	max_depth	Subsample	Learning_rate	Gamma	Cosample_bytree	Min_child_weight	Reg_alpha	Cosample_bylevel
1	289	4	0.3982	0.1245	2	0.4082	2.0175	2.5625	0.5318
2	451	4	0.7933	0.1225	2	0.3850	2.9332	0.3013	0.5811
3	397	5	0.8812	0.1421	1	0.1297	1.5058	1.2100	0.1645
4	258	6	0.8617	0.0749	0	0.2375	1.2412	2.4157	0.6032
5	362	10	0.7874	0.0448	2	0.6189	4.6050	0.001	0.5572
6	500	6	0.7235	0.0322	1	0.6725	4.0926	0.9204	0.4425
7	276	6	0.5948	0.1348	1	0.2414	3.4171	0.4942	0.4436
8	116	13	0.6446	0.1517	0	0.4655	2.0398	3.0595	1
9	269	9	0.2915	0.1449	0	0.6531	1.9371	2.4507	0.8564
10	113	6	0.7440	0.0735	0	0.5797	4.5738	0.9319	0.6483
11	87	3	0.1839	0.2	0	0.4114	1.0500	0.3872	0.7784
12	249	11	0.7843	0.0663	1	0.8085	1.00	0.6774	0.2351
13	183	8	0.5467	0.1496	1	0.3763	1.00	0.2118	0.12049
14	103	4	0.2045	0.1213	0	0.3993	2.3211	0.001	0.2581
15	165	3	0.6539	0.0746	0	0.4186	3.5303	0.5738	0.4267
16	465	14	0.2968	0.0347	2	0.8670	1.7779	0.1778	0.3557
17	134	5	0.2218	0.1549	0	0.4533	1.0475	0.0533	0.1
18	253	6	0.6964	0.0899	0	0.3160	3.7865	1.8783	0.5248
19	311	7	0.7335	0.1161	0	0.7701	1.9207	3.8390	0.5284
20	118	10	0.5790	0.0555	0	0.4242	4.5272	0.7323	0.1812

**Table 15:** List of hyperparameters for random forest find by SGO using Stalog dataset with preprocessing.

Sl.no	n_estimators	max_depth	min_samples_split	min_samples_leaf	max_features	bootstrap	Criterion	max_samples
1	105	34	5	1	4	True	entropy	0.5547
2	72	24	2	1	4	True	entropy	0.2593

**Table 16:** List of hyperparameters for XGBClassifie find by SGO using Statlog dataset with preprocessing.

Sl.no	n_estimators	max_depth	subsample	Learning_rate	gamma	Cosample_bytree	Min_child_weight	Reg_alpha	Cosample_bylevel
1	87	4	0.3317	0.06	3	0.2810	1.6375	0.001	0.236

Tables 13, 14, 15 and 16 summarize the hyperparameter configurations identified for RF and XGB Classifier using the SGO algorithm on the Statlog and Cleveland datasets, both with and without preprocessing. Table 11 presents two hyperparameter configurations for RF on the Statlog dataset without preprocessing. Both configurations yielded identical performance metrics: Acc. (0.9259), Prec. (1.00), Rec. (0.81), F1-S. (0.89), and RAUC Score (0.9278), demonstrating the robustness and reliability of the optimization process. Table 12 lists 20 hyperparameter configurations for XGB Classifier on the Statlog dataset without preprocessing. Each configuration achieved the same performance metrics: Acc. (0.9444), Prec. (1.00), Rec. (0.90), F1-S. (0.95), and RAUC Score (0.9524), underscoring the effectiveness of the optimization approach. On the Statlog dataset with preprocessing, Table 13 shows two hyperparameter settings for RF that produce the same results: Acc. (0.9524), Prec. (1.00), Rec. (0.90), F1-S. (0.95), and RAUC Score (0.9818). These results validate the reliability of the SGO algorithm in finding optimal configurations. Table 14 also displays an XGB Classifier hyperparameter configuration on the Cleveland dataset where it consistently achieves Acc. (0.9762) and Prec. (1.00). Rec. (0.95). F1-S (0.97). The RAUC Score (0.9750). The efficacy and resilience of the optimization process are further demonstrated by these outcomes.

## Generalizability of the proposed SGO-tuned ML model

The generalizability of the proposed SGO-tuned machine learning (ML) model is a crucial aspect, especially in the context of medical diagnosis where robustness across diverse patient populations is essential. Our model was validated using two publicly available and widely used heart disease datasets—Cleveland and Statlog—each with different data distributions and demographic characteristics. Consistently high performance across both datasets demonstrates the model’s ability to generalize well to different data sources.

Moreover, the integration of SGO for hyperparameter tuning contributes significantly to the model’s adaptability. By effectively balancing exploration and exploitation, SGO identifies optimal configurations that avoid overfitting to specific training data. This ensures that the learned patterns remain relevant and effective even when the model is exposed to new, unseen data.

To further assess generalizability, we employed k-fold cross-validation, which offers a reliable estimate of the model’s performance stability. The minimal variation in performance metrics across different folds underscores the robustness and consistency of the proposed approach.

Overall, the SGO-tuned ML framework exhibits strong potential for deployment in real-world clinical settings due to its high accuracy, reduced bias, and reliable generalization across diverse datasets. Future work may involve evaluating the model on additional multi-center or real-time clinical data to further confirm its applicability across broader populations.

## Conclusion and future directions

By combining the SGO algorithm with the RF and XGB Classifier models this study presents a scalable and efficient framework for the prediction of heart disease. The study methodically assesses how data preprocessing and SGO-based hyperparameter tuning affect model performance using the Cleveland and Statlog datasets. The findings show notable gains in several important metrics such as accuracy, precision, recall, and F1-score. The findings demonstrate the critical role that preprocessing plays in improving the quality of data with its effects varying according to the features of the dataset. Although preprocessing had varying effects on the Cleveland dataset it greatly improved Statlog’s performance on all metrics highlighting the necessity of dataset-specific preprocessing techniques. Furthermore SGO-tuned models achieved state-of-the-art acc. by consistently outperforming current techniques as well as durability. The SGO-tuned XGB Classifier achieves perfect prec. demonstrating how well these models balance sensitivity, specificity, and F1-S. of 0.97 on the Statlog dataset. These findings support the idea that SGO-driven hyperparameter tuning can improve classification models for challenging real-world problems. This research offers a dependable and scalable method for early heart disease prediction which significantly advances healthcare beyond technological advancements. The suggested approach promotes proactive disease management facilitates early diagnosis and may lower the death rate from cardiovascular disease.

## Future directions

The following topics could be investigated in future studies to improve the framework and increase its applicability.

- Expanding the dataset size using real-time clinical records.
- Applying the method to other diseases.
- Integrating temporal data for progression modeling.
- Using hybrid models combining DL with SGO-optimized components.
- Deploying the model in a real-time clinical decision support system (CDSS).

By demonstrating the effectiveness of SGO in optimizing predictive models, this study establishes the algorithm as a powerful tool for advancing ML applications in healthcare, here accurate and timely diagnostics play a crucial role in improving patient outcomes.

## Data Availability

The datasets used and/or analysed during the current study available from the corresponding author on reasonable request.

## Abbreviations

Acc: Accuracy
Prec: Precision
Rec: Recall
F1-S: F1-score
RAUC: ROC-AUC
M-Prec: Macro Precision
M-Rec: Macro Recall
M-F1-S: Macro F1-Score
W-Prec: Weighted Precision
W-Rec: Weighted Recall
W-F1-S: Weighted F1 Score
CM: Confusion matrix
